# Intracellular Bacteria Interfere with Dendritic Cell Functions: Role of the Type I Interferon Pathway

**DOI:** 10.1371/journal.pone.0099420

**Published:** 2014-06-10

**Authors:** Laurent Gorvel, Julien Textoris, Romain Banchereau, Amira Ben Amara, Wiwit Tantibhedhyangkul, Kristin von Bargen, Mignane B. Ka, Christian Capo, Eric Ghigo, Jean-Pierre Gorvel, Jean-Louis Mege

**Affiliations:** 1 Centre National de la Recherche Scientifique UMR 7278, IRD198, INSERM U1095, Aix-Marseille Université, Marseille, France; 2 Baylor Institute for Immunology Research, Dallas, Texas, United States of America; 3 Centre d'Immunologie de Marseille-Luminy (CIML), Aix-Marseille University, UM2, INSERM, U1104, CNRS, UMR7280, Marseille, France; 4 Department of Immunology, Faculty of Medicine, Siriraj Hospital, Mahidol University, Bangkok, Thailand; Institut de Pharmacologie et de Biologie Structurale, France

## Abstract

Dendritic cells (DCs) orchestrate host defenses against microorganisms. In infectious diseases due to intracellular bacteria, the inefficiency of the immune system to eradicate microorganisms has been attributed to the hijacking of DC functions. In this study, we selected intracellular bacterial pathogens with distinct lifestyles and explored the responses of monocyte-derived DCs (moDCs). Using lipopolysaccharide as a control, we found that *Orientia tsutsugamushi*, the causative agent of scrub typhus that survives in the cytosol of target cells, induced moDC maturation, as assessed by decreased endocytosis activity, the ability to induce lymphocyte proliferation and the membrane expression of phenotypic markers. In contrast, *Coxiella burnetii*, the agent of Q fever, and *Brucella abortus*, the agent of brucellosis, both of which reside in vacuolar compartments, only partly induced the maturation of moDCs, as demonstrated by a phenotypic analysis. To analyze the mechanisms used by *C. burnetii* and *B. abortus* to alter moDC activation, we performed microarray and found that *C. burnetii* and *B. abortus* induced a specific signature consisting of *TLR4*, *TLR3*, *STAT1* and interferon response genes. These genes were down-modulated in response to *C. burnetii* and *B. abortus* but up-modulated in moDCs activated by lipopolysaccharide and *O. tsutsugamushi*. This transcriptional alteration was associated with the defective interferon-β production. This study demonstrates that intracellular bacteria specifically affect moDC responses and emphasizes how *C. burnetii* and *B. abortus* interfere with moDC activation and the antimicrobial immune response. We believe that comparing infection by several bacterial species may be useful for defining new pathways and biomarkers and for developing new treatment strategies.

## Introduction

Dendritic cells (DCs) are specialized antigen-processing and -presenting cells that act as sentinels at the interface between innate and adaptive immunity [1]. In peripheral tissues, DCs are considered to be immature and are characterized by a high endocytic activity, the inability to activate T-cells and the intracellular expression of major histocompatibility complex (MHC) class II molecules [2], [3]. However, encounters with microorganisms or bacterial ligands, such as lipopolysaccharide (LPS), triggers the maturation of immature DCs [4], a process that is associated *in vivo* with their migration to secondary lymphoid organs where they interact with T-cells to orchestrate adaptive immune responses [1], [4].

Thus, the prevention of DC maturation and/or migration may be a relevant strategy for intracellular bacteria to avoid efficient immune responses, as illustrated by some examples of bacterial infections. *Salmonella enterica* serovar Typhimurium interferes with the migration of intestinal DCs and hinders antigen presentation via the ubiquitination and degradation of MHC class II molecules, which prevents bacterial killing [5], [6]. *Helicobacter pylori* replicates within autophagosomes and retains cytosolic MHC class II molecules; *H. pylori* also prevents interleukin (IL)-12 production and induces IL-10 secretion, thereby inhibiting the anti-microbial Th1 response [7], [8]. *Mycobacterium tuberculosis* interferes with Toll-like receptor (TLR) signaling through its binding to CD209, also known as the C-type lectin dendritic cell-specific intercellular adhesion molecule-3-grabbing non-integrin (DC-SIGN), blocking DC maturation and IL-12 production [9], [10]. Additionally, *Chlamydia trachomatis* and *C. psittaci* release molecules that delay antigen presentation by MHC class II molecules [11].

Unfortunately, most of these reports are based on the comparison of naïve DCs and DCs stimulated by only one type of microorganism [5]–[7], [9]–[11], and such an approach cannot identify the elements common to microorganisms sharing cellular targets and the relative level of DC impairment. Therefore, we selected four intracellular bacteria responsible for infectious diseases in humans and that exhibit distinct lifestyles within target cells. These four bacteria, namely, *Coxiella burnetii*, *Brucella abortus*, *Orientia tsutsugamushi* and *Tropheryma whipplei*, are known to target myeloid cells and to be pathogenic for humans: *O. tsutsugamushi*, the agent of scub typhus, lives in a cytosolic [12] compartment, whereas *C. burnetii*, the agent of Q fever, *B. abortus*, the agent of brucellosis, and *T. whipplei*, the agent of the Whipple's disease, are found in vacuolar compartments [13]–[15]. *O. tsutsugamushi* induces a strong inflammatory response, including the type I interferon (IFN) response, in monocytes [16] and macrophages [17]. It has been demonstrated *in vivo* that *O. tsutsugamushi* is preferentially located in dermal DCs from the eschars of patients with scrub typhus [18]. *T. whipplei* is a Gram-positive bacterium; this pathogen infects macrophages, inducing apoptosis [19] and an M2 non-microbicidal profile [20]. *C. burnetii*, the Gram-negative bacterium infects and resides within monocytes and macrophages. This bacterium induces an M1 inflammatory profile in monocytes and an M2 immunoregulatory profile [21] and also infects monocyte-derived DCs (moDCs) *in vitro*, appearing to prevent their maturation [22]. *B. abortus*, a Gram-negative bacterium, is known to infect macrophages and evade killing via the VirB type IV secretion system [23]. *B. abortus*, also infects DCs [24], and it has been shown that the bacterial btp1 protein partially inhibits the maturation of mouse bone marrow-derived DCs by inhibiting the Toll-like receptor (TLR)-2 and TLR4 pathways [24].

Here, we investigate the functions of moDCs infected with four intracellular bacterial pathogens to reappraise the concept of DC activation within the context of infectious diseases. Although *O. tsutsugamushi* induced moDC maturation, as did LPS, *C. burnetii* and *B. abortus* were less efficient. A transcriptional analysis identified an alteration of the IFN response pathway in moDCs infected with *C. burnetii* and *B. abortus*, whereas this pathway was fully operative in *O. tsutsugamushi*-infected moDCs. These results demonstrate that *C. burnetii* and *B. abortus* both interfere with the activation of human moDCs at the level of the IFN response, thus positioning this pathway in the pathogenesis of Q fever and brucellosis.

## Materials and Methods

### Ethics Statement

The experimental protocol was approved by the institutional animal care and use committee of Aix-Marseille University (décret N° 8 87–848, October 19, 1987, Paris). Blood was obtained from the Etablissement Français du Sang. It is a commercial product and the Ethics Committee agreement is not required.

### Bacteria

*O. tsutsugamushi* strain Kato (CSUR R163) was propagated in L929 cells, as previously described [12]. Briefly, highly infected cells were sonicated, or lysed using glass beads for *O. tsutsugamushi*, and centrifuged at 500× *g* for 10 minutes to remove the cell debris. The supernatants were collected and centrifuged at 10,000× *g* for 10 minutes to pellet the bacteria. The live bacteria were quantified using infected cell-counting units. *C. burnetii* organisms (RSA493 Nile Mile strain) were obtained by passage in BALB/c mice and culture in L929 cells. The concentration of organisms was determined by immunofluorescence using specific antibodies (Abs) and/or PCR using known concentrations of bacterial DNA. The quantification of organisms was performed as previously described [13]. Bacterial viability was assessed using the LIVE/DEAD BacLight bacterial viability kit (Invitrogen, Cergy Pontoise, France) [25]. *B. abortus* strain 2308 was grown on tryptic soy agar (Sigma-Aldrich, Saint-Quentin Fallavier, France) at 37°C for 4–5 days, as previously described [26]. The Twist-Marseille (CNCM I-2202) strain of *Tropheryma whipplei*, a bacterium known to live in macrophages, was cultured in HEL cells and purified as described elsewhere. The quantification of inocula was performed by measuring the percentage of infected cells by immunofluorescence, as previously described [19].

### Isolation and stimulation of moDCs

Blood from healthy donors was obtained from the Etablissement Français du Sang. Peripheral blood mononuclear cells (PBMCs) from buffy coats were recovered from the Ficoll-Hypaque interface after centrifugation at 700× *g* for 20 minutes. Monocytes and T-lymphocytes were isolated from PBMCs using magnetic beads coupled to Abs specific for CD14 and CD3, respectively, as described by the manufacturer (Miltenyi Biotec, Paris, France). The purity of the monocyte and T-cell preparations was assessed by flow cytometry and was greater than 98%. The monocytes were then incubated in RPMI 1640 containing 20 mM HEPES, 2 mM glutamine, 10% fetal calf serum (FCS) (Invitrogen), 0.1 ng/mL IL-4 and 1 ng/mL granulocyte-macrophage colony-stimulating factor (GM-CSF) (R&D Systems, Lille, France) for 7 days to obtain moDCs. The T-lymphocytes were frozen in a medium containing 10% dimethylsulfoxide. The moDCs were stimulated by 100 ng/mL *Escherichia coli* LPS (Sigma-Aldrich) or 20 bacterial cells per moDC. For the microarray experiments, 300 *B. abortus* cells per moDC were used to maximize the infection of moDCs. In some experiments, *C. burnetii* LPS, kindly provided by Dr. R. Toman) [13] and *B. abortus* LPS [27] were used at 1 µg/mL.

### Functional activity of moDCs

The endocytic activity of moDCs was examined by measuring the uptake of fluorescein isothiocyanate (FITC)-coupled albumin. moDCs were cultured in 6-well plates (10^6^ cells per well) in RPMI 1640 containing 10% FCS and stimulated with pathogens, or LPS as a control of maturation, for 24 hours at 37°C. After washing in phosphate-buffered saline (PBS), the moDCs were incubated with 20 µg/mL FITC-coupled albumin (Invitrogen) for 1 hour; the moDCs were then washed, scraped and fixed in 3% paraformaldehyde (PFA) for 15 minutes. The cell fluorescence was analyzed by flow cytometry using the FACS Canto II flow cytometer (BD Biosciences, Le Pont de Claix, France). The mean fluorescence intensity (MFI) was recorded, and the results are expressed relative to the fluorescence of unstimulated DCs.

The ability of moDCs to induce T-cell proliferation was investigated as follows. moDCs (2×10^5^/mL) were cultured in 12-well plates and stimulated with bacteria or LPS for 24 hours. Autologous T-lymphocytes (10^6^ cells/mL) were labeled in 500 µL of PBS containing 10% of bovine serum albumin and 10 µM carboxyfluorescein diacetate succinimidyl ester (CFSE) (Invitrogen) for 15 minutes. The T-lymphocytes were co-cultured with moDCs (ratio of 5∶1) for 5 days, and the fluorescence of the T-lymphocytes was analyzed by flow cytometry. The T-lymphocyte proliferation was determined as the percentage of cells with decreased CFSE fluorescence. The results are expressed relative to the fluorescence of unstimulated moDCs.

### Expression of DC maturation markers

moDCs were stimulated in 6-well plates (10^6^ cells) in RPMI 1640 containing 10% FCS with bacterial pathogens or LPS extracted from *E. coli*, *C. burnetii* or *B. abortus* for 24 hours. The moDCs were scraped, centrifuged at 400× *g* for 5 minutes and incubated with conjugated with FITC-CD80, phycoerythrin (PE)-CD83, PE-CD86 and FITC-HLA-DR for 30 minutes. The moDCs were fixed with 3% PFA for 20 minutes, washed and incubated in 500 µL of PBS prior to the flow cytometry analysis. The fluorescence of 20,000 cells per sample was quantified using the FACS Canto II flow cytometer (BD Biosciences). The results are expressed as the fold change (FC) defined as the MFI of stimulated moDCs and the MFI of unstimulated moDCs.

### Microarrays

moDCs (5×10^6^ cells per well) were plated in 6-well plates and stimulated with bacteria (MOI of 20∶1, except for *B. abortus* at an MOI of 300∶1) or LPS for 6 hours, and total RNA was then extracted using the RNeasy minikit (Qiagen, Courtaboeuf, France) and DNase treatment. This study utilized the 4X44k Human Whole Genome microarrays (Agilent Technologies, Les Ulis, France), representing 44,000 probes, as recently described [25]. Reverse transcription, sample labeling and hybridization were performed according to the protocols specified by the manufacturer (One-Color Microarray-Based Gene Expression Analysis). Three samples per experimental condition were included in the analysis. The slides were scanned at a 2-µm resolution with a G2505C DNA microarray scanner (Agilent Technologies). The data were generated in compliance with the Minimum Information About a Microarray Experiment (MIAME) guidelines and were deposited in the Gene Expression Omnibus of the National Center for Biotechnology Information (accession number: GSE49016).

Image analysis and intra-array signal corrections were performed using Agilent Feature Extractor Software A.9.1.3. The microarray data analysis was performed using R (v.3.0.1) and the Bioconductor software suite, and the raw data were filtered and normalized using the Agi4x44PreProcess library. Unsupervised and supervised analyses were performed using hierarchical clustering and a principal component analysis (PCA) (*made4* library [28] and Linear Models for Microarray Analysis (*limma* library) [29]). Genes were considered to be differentially expressed if the False Discovery Rate (FDR) [30] was below 0.1% and the absolute fold change (FC) was above 2.0. The core program of the moDCs induced by *O. tsutsugamushi*, *C. burnetii* and *B. abortus* was annotated using the DAVID annotation tool [31] and Kyoto Encyclopedia of Gene and Genome (KEGG) pathways [32]. The integration and rendering of the pathway-based data were performed using the library *pathview* [33].

### Real-time RT-PCR

The genes in the IFN pathway that were identified by the microarray as being modulated were selected and validated by quantitative real-time reverse transcriptase PCR (qRT-PCR), as previously described [25]. In brief, reverse transcription of 150 ng of RNA was performed using an Moloney murine leukemia virus (MMLV) RT kit (Invitrogen). The first-strand cDNA was obtained using oligo(dT) primers and MMLV RT (Invitrogen), and the qPCR experiments were performed using SYBR Green Fast Master Mix (Roche Diagnostics, Meylan, France) and an ABI7900 Fast Real-Time PCR System (Life Technologies). The primers were designed using Primer3. The results were normalized to the housekeeping gene β-actin and are expressed as the median of fold change (FC)  = 2^−ΔΔCt^, where ΔΔCt  =  (Ct_Target_ − Ct_Actin_)_stimulated_ − (Ct_Target_ − Ct_Actin_)_unstimulated_, as previously described [34].

### Phosphorylation of p38

moDCs were stimulated with 1 µg/mL *E. coli* LPS or pathogens for different periods. The activation of the mitogen-activated protein kinase (MAPK) p38 was determined by immunoblotting using Abs specific for phospho-p38 (1/1000 dilution, Cell Signaling). The expression of activated p38 was quantified by densitometric scanning after normalization with structural p38. The results are expressed in relative intensities.

### IFN-β immunoassay

moDCs (10^5^ cells/mL) plated in 24-well plates were stimulated with pathogens or different concentrations of *E. coli* LPS for 16 hours, and the supernatants were centrifuged at 1000×*g* for 10 minutes and frozen at −80°C. The Verikine Human IFN-β ELISA kit (R&D Systems) was used to determine IFN-β production by the moDCs; the sensitivity of the kit was 3 pg/ml, and the inter- and intra-assay specificity was ≤ 8%.

### Statistical analysis

The results are expressed as the means ± SEM and were compared using Student's *t*-test. When the comparisons involved more than two conditions, the analysis was made with ANOVA test. *P* values less than 0.05 were considered significant.

## Results

### *C. burnetii* and *B. abortus* partly induce moDC maturation

**Figure 1:**
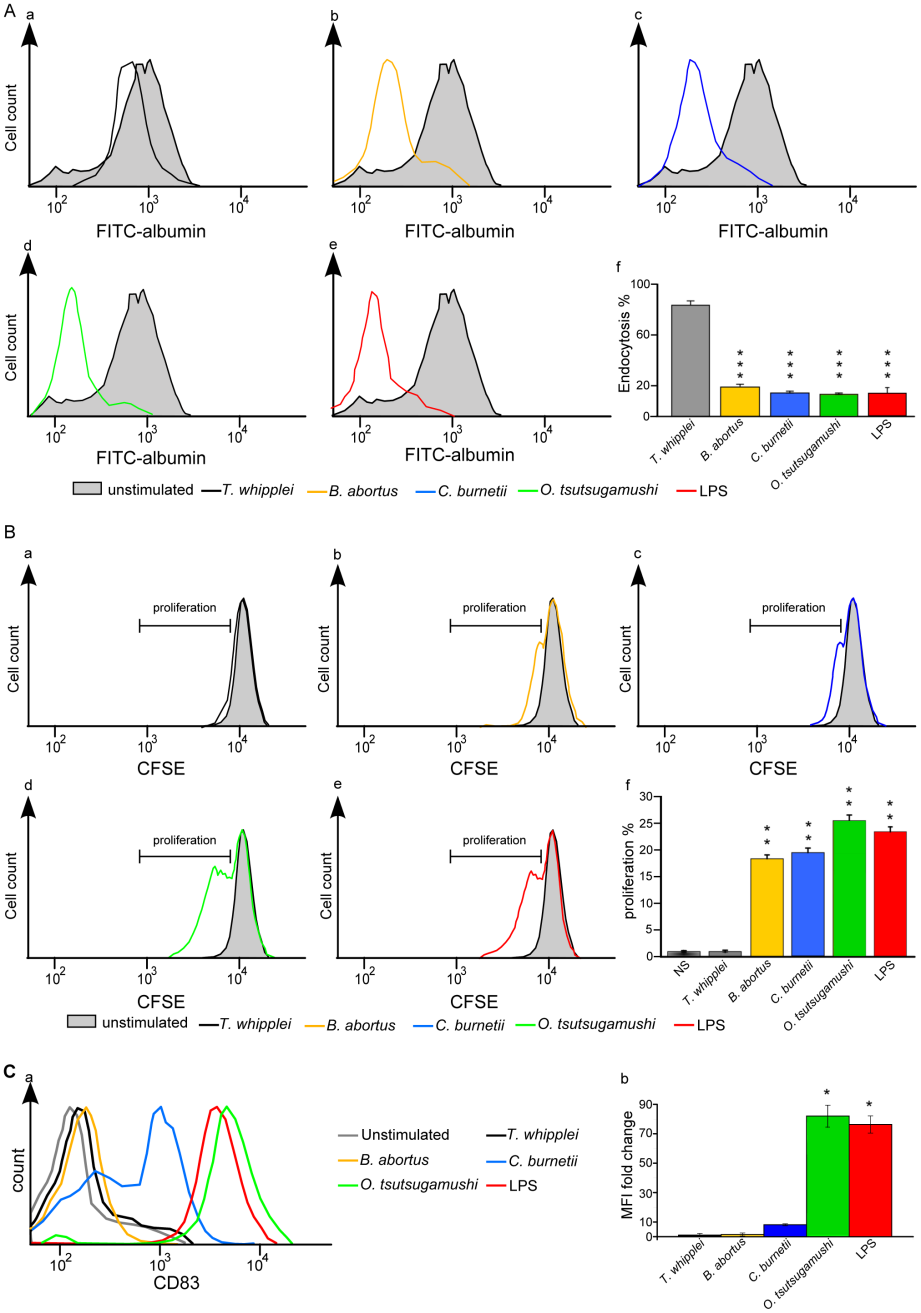
Functional study of moDC maturation. **A**, moDCs were stimulated with *T. whipplei* (a), *B. abortus* (b), *C. burnetii* (c), *O. tsutsugamushi* (d) or *E. coli* LPS (e) for 24 hours and then incubated with FITC-albumin (1 mg/mL) for 1 hour. The fluorescence intensities of unstimulated moDCs and stimulated moDCs were determined by flow cytometry. The results are expressed as the ratio of stimulated moDC fluorescence over unstimulated moDC fluorescence (f). They represent the mean ± SD of three independent experiments. ***p<0.001 represents the comparison of stimulated moDCs vs unstimulated DCs using Student's t-test. **B**, moDCs stimulated with *T. whipplei* (a), *B. abortus* (b), *C. burnetii* (c), *O. tsutsugamushi* (d) or *E. coli* LPS (e) were co-cultured with CFSE-labeled autologous T-lymphocytes for 5 days. The gating strategy used to determine lymphocyte proliferation is shown for each stimulation (a–e). The results are expressed as the percentage of new T-cells (cells with decreased fluorescence intensity) in the total T-cell population (f). They represent the mean ± SD of three independent experiments. **p<0.02 represents the comparison of stimulated moDCs vs unstimulated DCs using Student's t-test. **C**, The membrane expression of CD83 by moDCs incubated with bacterial pathogens or LPS for 24 hours was determined by flow cytometry using PE-coupled anti-CD83 Abs (a). The histograms are representative of three different experiments. The FC of MFI values after stimulation was compared to unstimulated condition (b). *p<0.03 represents the comparison of stimulated moDCs vs unstimulated DCs using Student's t-test.

We investigated the maturation of moDCs in response to the cytoplasmic bacterial pathogen *O. tsutsugamushi* and three vacuolar bacteria, *C. burnetii*, *B. abortus* and *T. whipplei*, using three criteria: the loss of endocytosis, the ability to induce lymphocyte proliferation and the expression of CD83. First, the endocytosis activity of the moDCs stimulated with *T. whipplei* was not different (80% ± 8%) from that of unstimulated cells (**Figure 1A** **, a and f**). In contrast, the endocytosis activity of the moDCs stimulated with *B. abortus C. burnetii* and *O. tsutsugamushi* was dramatically reduced compared to the control moDCs (21% ± 3%, 18% ± 2% and 17% ± 5%, respectively, **Figure 1A** **, b–d and f**). Note that the endocytosis activity of the moDCs stimulated with *B. abortus*, *C. burnetii* and *O. tsutsugamushi* was similar to that observed in moDCs stimulated with *E. coli* LPS (18% ± 2%, **Figure 1** **, e and f**). Second, the ability of moDCs to induce proliferation of autologous T-cells after moDC infection was assessed by flow cytometry. We found that *T. whipplei* was unable to affect T-cell proliferation (**Figure 1B** **, a and f**), as recorded by CFSE fluorescence. In contrast, *B. abortus*, *C. burnetii* and *O. tsutsugamushi* induced a significant T-cell proliferation compared to the unstimulated moDCs (**Figure 1B** **, b, c, d and f**): T-cell proliferation was similar to that observed in LPS-stimulated moDCs (**Figure 1B** **, e and f**). Third, we found that the measurement of CD83 expression was more discriminative than the two other features of moDC maturation. *O. tsutsugamushi* induced a strong CD83 expression (when compared to the unstimulated condition, the FC of MFI values was >80) that was similar to that induced by LPS (MFI FC>75). In contrast, CD83 was only weakly expressed in the *C. burnetii*-stimulated moDCs (MFI FC<10) and was absent in the moDCs stimulated by *B. abortu*s or *T. whipplei* (**Figure 1C** **, a and b**). Taken together, these results demonstrate that *O. tsutsugamushi* was fully efficient to induce the maturation of moDCs whereas *C. burnetii* and *B. abortus* partly induce moDC maturation.

### *B. abortus* and *C. burnetii* did not share the same ability to activate moDCs

**Figure 2:**
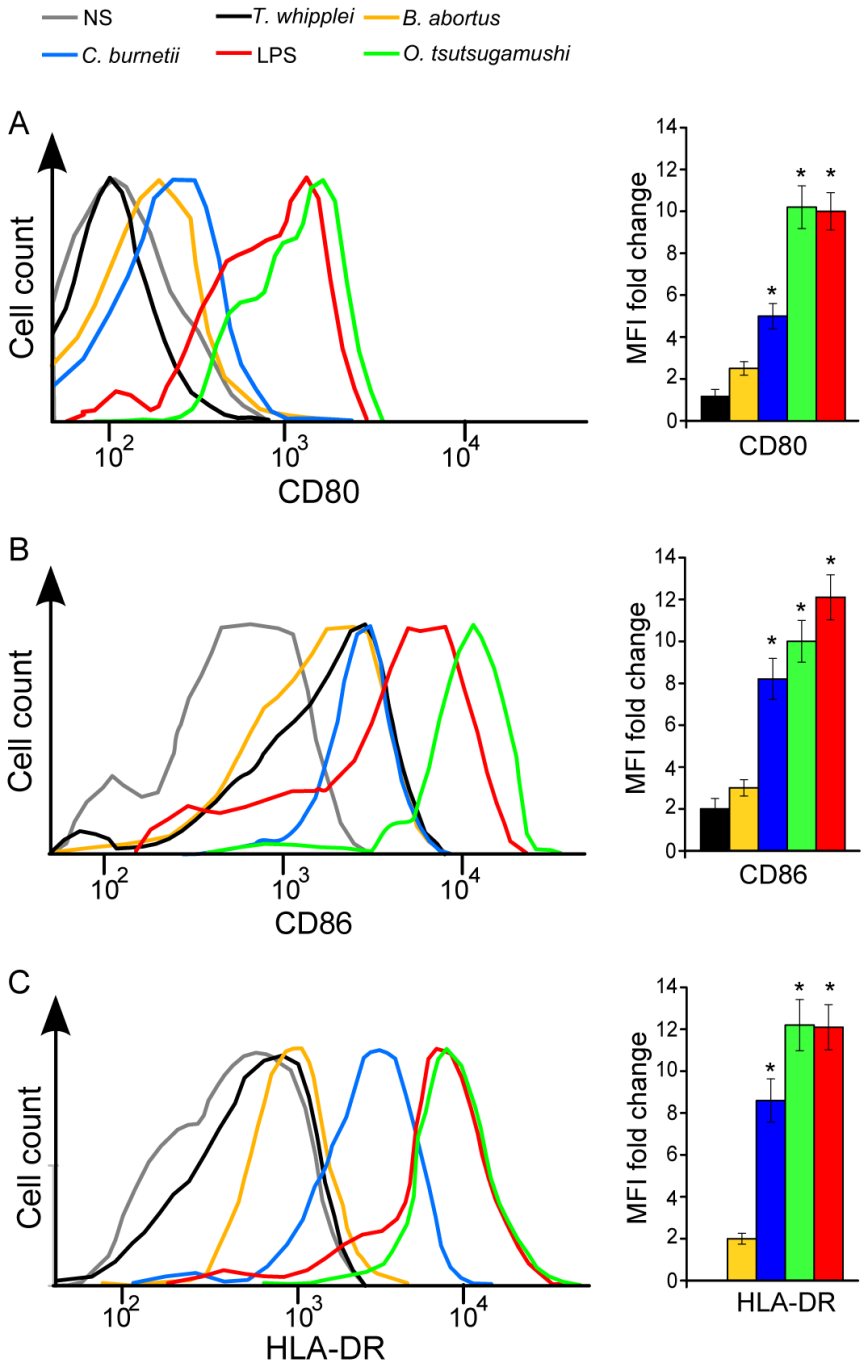
Phenotypic study of moDC maturation. moDCs were stimulated with bacterial pathogens or *E. coli* LPS for 24 hours. The cells were then incubated with FITC-coupled anti-CD80 (A), PE-coupled anti-CD86 (B) and FITC-coupled anti-HLA-DR (C) Abs for 30 minutes and analyzed by flow cytometry. The histograms are representative of three different experiments. The bar charts represent the MFI FC values of stimulated moDCs compared with unstimulated moDCs. *p<h0.05.

As the expression of CD83 was differently affected by pathogens, we addressed whether these bacteria affected other features of moDC maturation, including the membrane expression of such costimulation molecules as CD80 and CD86 and that of HLA-DR, a MHC class II molecule. In agreement with its ability to induce the maturation of moDCs, we found that *O. tsutsugamushi* markedly increased the expression of CD80 (MFI FC of 10), CD86 (MFI FC of 10) and HLA-DR (MFI FC of 12), as did LPS. In contrast, *C. burnetii* only slightly affected the membrane expression of CD80 (MFI FC of 5), CD86 (MFI FC of 8) and HLA-DR (MFI FC of 8); *B. abortus* also poorly affected the expression of CD80 (MFI FC of 2) and CD86 (MFI FC of 4) but not that of HLA-DR. It should be noted that *T. whipplei* had a limited effect on the expression of CD80, CD86 and HLA-DR (**Figure 2**). Taken together, these results emphasize the profound deficiency of *B. abortus*-stimulated moDCs and the relative alteration of *C. burnetii*-stimulated moDCs to express activation markers.

### Microarray analysis reveals specific responses to intracellular bacteria

**Figure 3:**
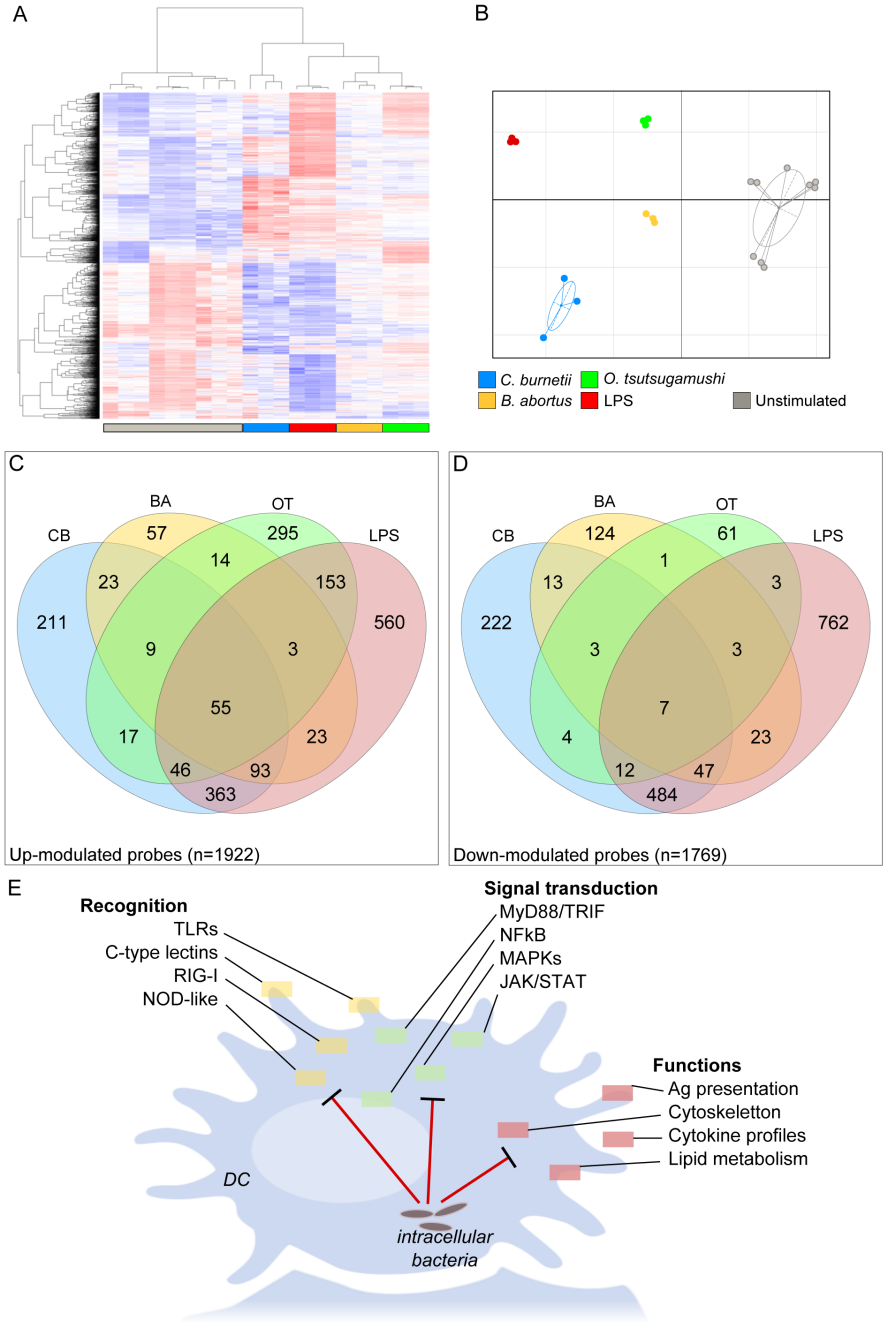
Transcriptional program of stimulated moDCs. moDCs were stimulated with *B. abortus*, *C. burnetii*, *O. tsutsugamushi* or *E. coli* LPS for 6 hours. RNA was extracted, and a microarray was performed. **A**, A heatmap representation of the 3,610 modulated probes in stimulated moDCs compared to unstimulated moDCs (NS). The probes are shown in the rows and the samples in the columns. The expression levels are color coded from blue to red. **B**, Graphical representation of the samples based on the correspondence analysis of the modulated probes. The samples are colored according to the stimulus. **C**–**D**, Venn diagrams highlighting the core and specific signatures induced in moDCs stimulated with LPS and pathogens, showing up-modulated probes (C) and down-modulated probes (D). **E**, Summary of the pathways in moDCs modulated by intracellular bacteria. Ba: *B. abortus*; Cb: *C. burnetii*; Ot: *O. tsutsugamushi*.

We used a systems biology approach to investigate the mechanisms leading to the different patterns of activation in response to LPS, *C. burnetii*, *B. abortus* and *O. tsutsugamushi* and found that the stimulated moDCs were present in a cluster that was different from that of the unstimulated moDCs (**Figure 3A**), with 3,610 modulated probes (2,609 distinct genes). We removed *T. whipplei*-stimulated moDCs from the analysis because we were unable to identify a transcriptional signature that was not different from background analysis. Hierarchical clustering and PCA also discriminated the global transcriptional signature of each bacterium; note that the global signatures induced by *O. tsutsugamushi* and LPS were largely different from those induced by *C. burnetii* and *B. abortus* (**Figure 3B**). Using Venn diagrams, we identified a core response to LPS and pathogens: this core response included 62 genes (55 up-modulated and 7 down-modulated). We also identified specific signatures for LPS and each bacterium (**Figure 3C and 3D**).

We then analyzed the gene pathways stimulated by LPS and bacterial pathogens. Among the 2,609 genes modulated, we found that 469 genes (20%) were implicated in several canonical immune response pathways (extracted from the KEGG database, **Table 1**), such as recognition pathways (the TLR signaling pathway, NOD-like signaling pathway, RIG-I-like signaling pathway and cytosolic DNA-sensing pathway), signal processing (the Jak-STAT and MAPK signaling pathways) or functions (cytokine-cytokine receptor interaction, chemokine signaling pathway and apoptosis) (**Figure 3E**). The analysis of each pathway revealed numerous differences, which were specific for each bacterium (**Results S1**). Taken together, these results showed that, in addition to a core program related to the immune response, each microorganism is able to specifically modulate this response.

**Table 1:** KEGG pathways identified in stimulated moDCs.

KEGG pathway name (#ID)	Number of genes	Corrected *P* value
Cytokine-cytokine receptor interaction (#04060)	99	2.0×10^−18^
Jak-STAT signaling pathway (#04630)	51	2.0×10^−6^
RIG-I-like receptor signaling pathway (#04622)	30	4.5×10^−6^
NOD-like receptor signaling pathway (#04621)	27	8.5×10^−6^
Cytosolic DNA-sensing pathway (#04623)	25	8.3×10^−6^
Chemokine signaling pathway (#04062)	55	1.2×10^−5^
Toll-like receptor signaling pathway (#04620)	35	3.5×10^−5^
Apoptosis (#04210)	29	6.8×10^−4^
P53 signaling pathway (#04115)	22	9.8×10^−3^
MAPK signaling pathway (#04010)	58	3.3×10^−2^
Focal adhesion (#04510)	46	3.4×10^−2^

moDCs were stimulated with *E. coli* LPS or bacterial pathogens for 6 hours. RNAs were extracted, and microarrays were performed. The modulated genes were analyzed using the KEGG database. The KEGG pathways, the number of modulated genes and *P* values are shown.

### *C. burnetii* and *B. abortus* interfere with the type I IFN response pathway

**Figure 4:**
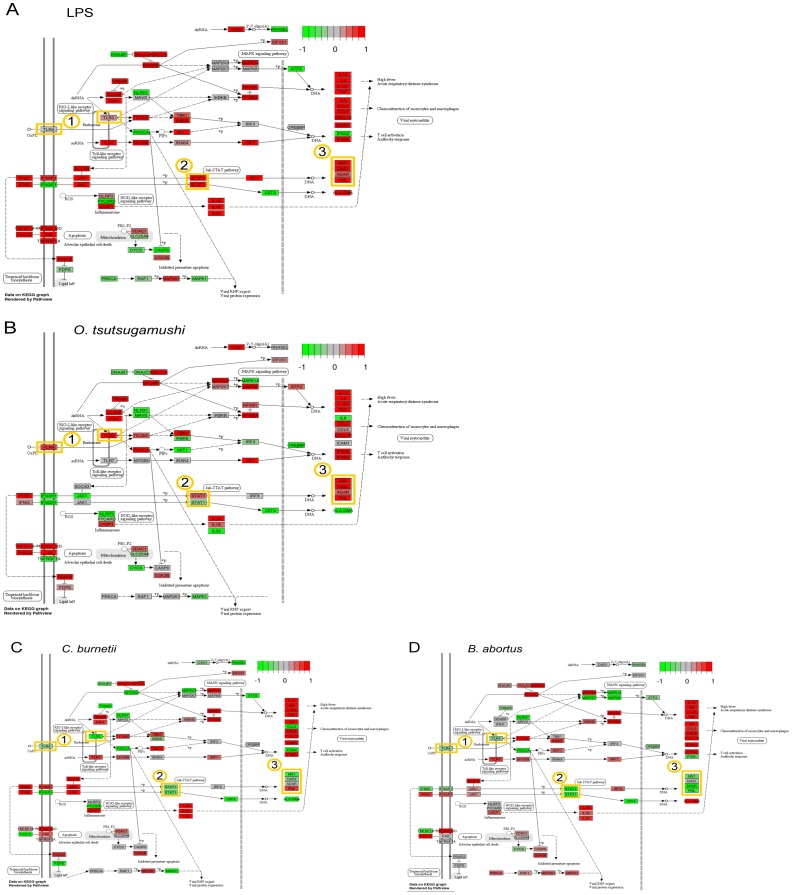
Activation of the type I IFN response pathway by bacteria. The KEGG pathway hsa05164 of influenza response was modified to represent the type I IFN response. The genes depicted on this pathway are colored according to the modulation induced by *E. coli* LPS (A), *O. tsutsugamushi* (B), *C. burnetii* (C) or *B. abortus* (D).

Although each microorganism elicited a specific signature in moDCs, the *C. burnetii*- and *B. abortus*-stimulated responses were clearly distinct from the *O. tsutsugamushi*- and LPS-stimulated responses, suggesting major differences between those agonists that induced full maturation and the others. By selecting the genes that were up-modulated by LPS and *O. tsutsugamushi* and down-modulated by *C. burnetii* and *B. abortus*, we identified a series of genes implicated in apoptosis regulation, innate immune response and type I IFN “antiviral” response (**Table 2**). A set of genes involved in the IFN pathway and that were down-modulated in response to *C. burnetii* and *B. abortus* was confirmed by qRT-PCR (**Table S1**). In addition, a comparison of the KEGG “Influenza” pathway (which summarizes the main modules of the type I IFN response pathway) in the stimulated moDCs highlighted three nodes that were down-modulated by *C. burnetii* and *B. abortus* (**Figure 4**), corresponding to the genes encoding TLR3/TLR4, the transcription factor Stat1 and such antiviral response genes as *MX1, MX2, OAS1, IFI44* and *ADAR*.

**Table 2:** Genes differentially expressed by bacterial pathogens.

Symbol	*C. burnetii*	*B. abortus*	*O. tsutsugamushi*	LPS
ATP10A	0.45	0.85	1.20	6.00
BRIP1	0.48	1.08	6.85	13.51
CCL2	0.38	1.80	3.47	5.01
FAM59A	0.26	1.45	3.65	2.89
GMPR	0.24	0.78	2.10	6.24
HERC5	0.47	1.30	14.08	36.91
HSH2D	0.39	0.91	0.99	6.02
IFI44	0.45	0.97	3.63	11.52
MX1	0.46	0.98	2.48	6.43
MX2	0.49	0.95	1.63	13.35
OR52K3P	0.31	0.80	3.58	5.28
P2RY6	0.34	0.91	0.96	2.06
SAMD9	0.43	0.91	3.55	5.30
SH2D1B	0.49	1.24	3.32	4.75
SIX1	0.40	1.19	3.73	3.43
TRIL	0.40	0.96	3.32	9.13
USP18	0.46	1.10	4.16	13.52
XAF1	0.50	1.00	1.27	5.69

moDCs were stimulated with bacterial pathogens or *E. coli* LPS for 6 hours. RNAs were extracted, and microarrays were performed. The genes that were up-modulated by *O. tsutsugamushi* and LPS and down-modulated by *C. burnetii* and *B. abortus* are listed in alphabetic order. The values of FC are presented and IFN response genes are underlined.

**Figure 5:**
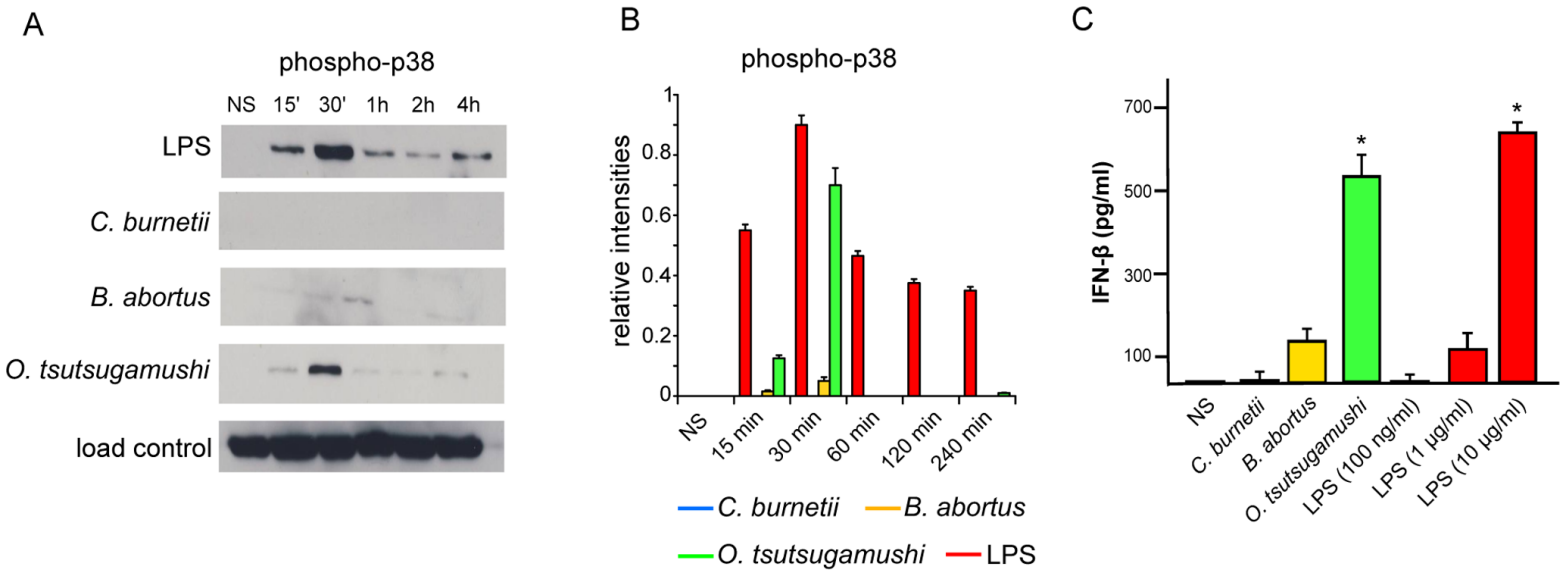
p38 phosphorylation and IFN-β release. **A**, moDCs were stimulated with bacterial pathogens or *E. coli* LPS for different durations, ranging from 15 minutes to 4 hours, and immunoblotting was used to assess MAPK p38 phosphorylation. **B**, Densitometric scanning was used to quantify phosphorylation changes of p38 and the results are the mean ± SD of three experiments. **C**, moDCs were stimulated with bacterial pathogens or different concentrations of LPS for 16 hours. The release of IFN-β by moDCs was determined by ELISA. The results are expressed in pg/mL and represent the mean ± SD of three independent experiments.*p<0.03.

The transcriptional alterations in the moDCs stimulated by *C. burnetii* and *B. abortus* were associated with defective activation of the MAPK p38. Hence, the phosphorylation of p38, 15 and 30 minutes after *C. burnetii* and *B. abortus* stimulation, was lower that observed in the moDCs stimulated with LPS or *O. tsutsugamushi* (**Figure 5** **, A and B**). The transcriptional alterations induced by *C. burnetii* and *B. abortus* were also associated with defective IFN-β production Indeed, *C. burnetii* was unable to induce the release of IFN-β, a type I IFN, from the moDCs. Similarly, *B. abortus*-stimulated moDCs released low levels of IFN-β. In contrast, *O. tsutsugamushi* induced the release of high levels of IFN-β from the moDCs. LPS, which induces type I IFN genes, induced the release of IFN-β in a dose-dependent manner (**Figure 5C**). These results demonstrate that the defective maturation of moDCs in response to *C. burnetii* and *B. abortus* is associated with defective gene expression within the type I IFN pathway, defective p38 activation and defective IFN-β production.

## Discussion

This study addressed how intracellular bacteria responsible for human infectious diseases affect the response of moDCs, thereby creating an immune environment that is favorable to bacterial survival. We selected two intraphagosomal bacteria, *C. burnetii* and *B. abortus*, and one intracytosolic microorganism, *O. tsutsugamushi*. Although each pathogen has specificity in metabolism, virulence and replication rate, these bacteria share the ability to target myeloid cells [17], [18], [21], [22], [24]; the former two cause infectious diseases with a risk of chronic evolution, whereas the latter is responsible for acute infections. Such observations suggest that the immune mechanisms, including DC activation, induced by *C. burnetii* and *B. abortus* are different from those induced by *O. tsutsugamushi*. The combined use of functional tests of maturation and the measurement of activation marker membrane expression revealed that the responses of moDCs to *O. tsutsugamushi* were similar to those induced by LPS, corresponding to DC maturation. It has also been reported that *O. tsutsugamushi* activates moDCs and drives the proliferation of CD4^+^ T-cells [12], [18]. The combined approach in the present study showed that *C. burnetii* and *B. abortus* partly induce the maturation of moDCs, with *B. abortus* being less efficient than *C. burnetii*. These findings are consistent with previous reports showing that *C. burnetii* partially impairs the maturation of moDCs [22] and that *B. abortus* inhibits DC maturation in a murine model of infection [24]. Our results demonstrate that the use of whole functional tests, including the loss of endocytic activity and T-cell proliferation, were not sufficiently accurate to discriminate among the responses of DCs to bacteria. These assays might also lead to erroneous conclusions, as *O. tsutsugamushi, C. burnetii* and *B. abortus* share the ability to induce the loss of endocytosis and T-cell proliferation. The discrepancy between the expression of membrane antigens and functional tests of maturation has been described in *Mycobacterium tuberculosis* infection. Indeed, the subsets of DCs that are infected at high frequency poorly stimulate *M. tuberculosis* antigen-specific CD4^+^ T-cells, despite the expression of surface MHC class II molecules and costimulatory molecules [35]. Alternatively, the production of IL-2 and IL-15 by DCs in response to infectious stimuli may be involved in T-cell proliferation [36], [37]. We analyzed the microarray data from stimulated moDCs and found that the *IL15* gene was overexpressed in response to *C. burnetii, O. tsutsugamushi* and LPS. Similarly, the *IL2* gene was upmodulated in response to *C. burnetii* and LPS while the *IL2RA* gene was overexpressed in response to *O. tsutsugamushi, C. burnetii* and *B. abortus* (**Table S2**). These findings are consistent with the hypothesis that the expression of IL-2 and IL-15 pathways is sufficient to induce T-cell proliferation whereas the modulation of membrane molecules requires additional signals.

As our phenotypic assays identified defective moDC maturation in response to *C. burnetii* and *B. abortus*, we used a high-throughput transcriptional approach to characterize the underlying mechanisms. We used the *E. coli* LPS signature and the signature induced by *O. tsutsugamushi* as positive controls of maturation. The absence of LPS from the cell wall of *O. tsutsugamushi* [17] rules out wall LPS as stimulating *O. tsutsugamushi*-induced full DC maturation, which is emphasized by the failure of LPS-expressing *C. burnetii* and *B. abortus* to fully induce the maturation of DCs. Nevertheless, the LPS of these bacteria are atypical. *C. burnetii* LPS is poorly endotoxinic and exhibits a specific molecular composition with two unusual sugars: virenose and dihydroxystreptose [13]. The treatment of moDCs with *C. burnetii* LPS up-regulated the expression of CD86 and HLA-DR but not that of CD80 and CD83, suggesting that bacterial LPS contributes to the phenotype of *C. burnetii*-stimulated DCs (**Figure S1**). However, these results do not support the hypothesis of Shannon *et al.* that *C. burnetii* LPS interferes with *C. burnetii*-activated DCs [22]. We found that *B. abortus* LPS induced an moDC phenotype similar to that induced by the entire bacterium, as only CD80 and CD86 were expressed at the moDC membrane (**Figure S1**), suggesting that *B. abortus* LPS is unable to fully activate moDCs. Indeed, *B. abortus* LPS might act along with the btp1 protein [24] to prevent the proper DC-driven immune response. Here, we confirm that the LPSs from *C. burnetii* and *B. abortus* are not sufficient to activate moDC responses.

We focused on the moDC responses induced by *C. burnetii* and *B. abortus*. The activation of the MAPK p38 was defective in response to *C. burnetii* and *B. abortus*. The defective activation of p38 by *C. burnetii* has been reported in macrophages and related to the escape of bacteria to degradative compartments [13]. The modulation of p38 by *Brucella* sp. is debated. The LPS O chain from *Brucella* sp. restricts the activation of p38 [38] and the intracellular replication of *Brucella melitensis* is mainly dependent of p38 [39]. In contrast, *B. abortus* activates p38 in murine astrocytes [40]. The transcriptional signature induced by *C. burnetii* and *B. abortus* compared to that induced by *O. tsutsugamushi* and LPS was composed of a series of down-modulated genes. The comparison of the KEGG “Influenza” response pathway led to the identification of nodes targeted by *C. burnetii* and *B. abortus* to down-modulate *TLR4*/*TLR3*, *STAT1* and type I IFN-responsive genes. We recently explored the transcriptional datasets that are deposited in public repertories, such as the Gene Expression Omnibus, and we focused the main transductional pathways that lead to the type I IFN response during bacterial infections. We found that the type I IFN pattern induced by vacuolar bacteria is completely different from the signatures induced by viruses and cytosolic bacteria [41]. The IFN response pathway has been described in transcriptomics analysis of tuberculosis in which it remains silent in latent tuberculosis and was fully active in patients with active tuberculosis [42]. As *C. burnetii* and *B. abortus* share the ability to cause a chronic evolution of disease and bacterial persistence, such a strategy may be operative for their persistence. *C. burnetii* was unable to stimulate the release of IFN-β, and *B. abortus* only induced the release of low levels of IFN-β compared to *O. tsutsugamushi*. It has been recently demonstrated that *B. abortus* induced the production of IFN-β by moDCs in mice [43]. The recognition of *O. tsutsugamushi* by cytosolic sensors and the activation of the type I IFN signaling pathway [44] may be related to the ability of *O. tsutsugamushi* to escape phagosomes and enter the cytosol [45], as found for *Francisella tularensis* [46] and *Listeria monocytogenes* [47], [48]. IFN-β was also reported to be produced by monocytes and macrophages in response to *O. tsutsugamushi* [16], [17], suggesting that the intracellular lifestyle of *O. tsutsugamushi* is critical for the production of type I IFN.

In conclusion, we showed that intracellular bacteria, such as *C. burnetii* and *B. abortus*, are able to modulate the maturation of moDCs. The classical functional tests of DC maturation are not sufficiently accurate to detect impaired DC responses. However, using a systems biology approach, we found that *C. burnetii* and *B. abortus* dampen the type I IFN response. This effect may lead to inefficient immune response and bacterial persistence within the host, conferring a critical role for the type I IFN pathway in antibacterial immunity.

## Supporting Information

Figure S1 **Phenotypic study of moDC maturation.** moDCs were stimulated with *C. burnetii*-LPS, *B. abortus*-LPS or *E. coli*-LPS for 24 hours. The cells were then incubated with FITC-coupled anti-CD80, PE-coupled anti-CD83, PE-coupled anti-CD86 and FITC-coupled anti-HLA-DR Abs for 30 minutes and analyzed by flow cytometry. The curves are representative of three different experiments.
(TIF)

Table S1 **qRT-PCR validation of genes involved in the IFN pathway.**
(DOC)

Table S2 **IL-15 and IL-12-associated genes.**
(DOC)

Results S1 **Pathway analysis of transcriptional modulation of moDC by intracellular bacteria.**
(PDF)
