# Supplementary figures and images for: Intracellular Bacteria Interfere with Dendritic Cell Functions: Role of the Type I Interferon Pathway
Source: PLoS One. 2014 Jun 10;9(6):e99420. doi: 10.1371/journal.pone.0099420 (PMC4051653; doi:10.1371/journal.pone.0099420)

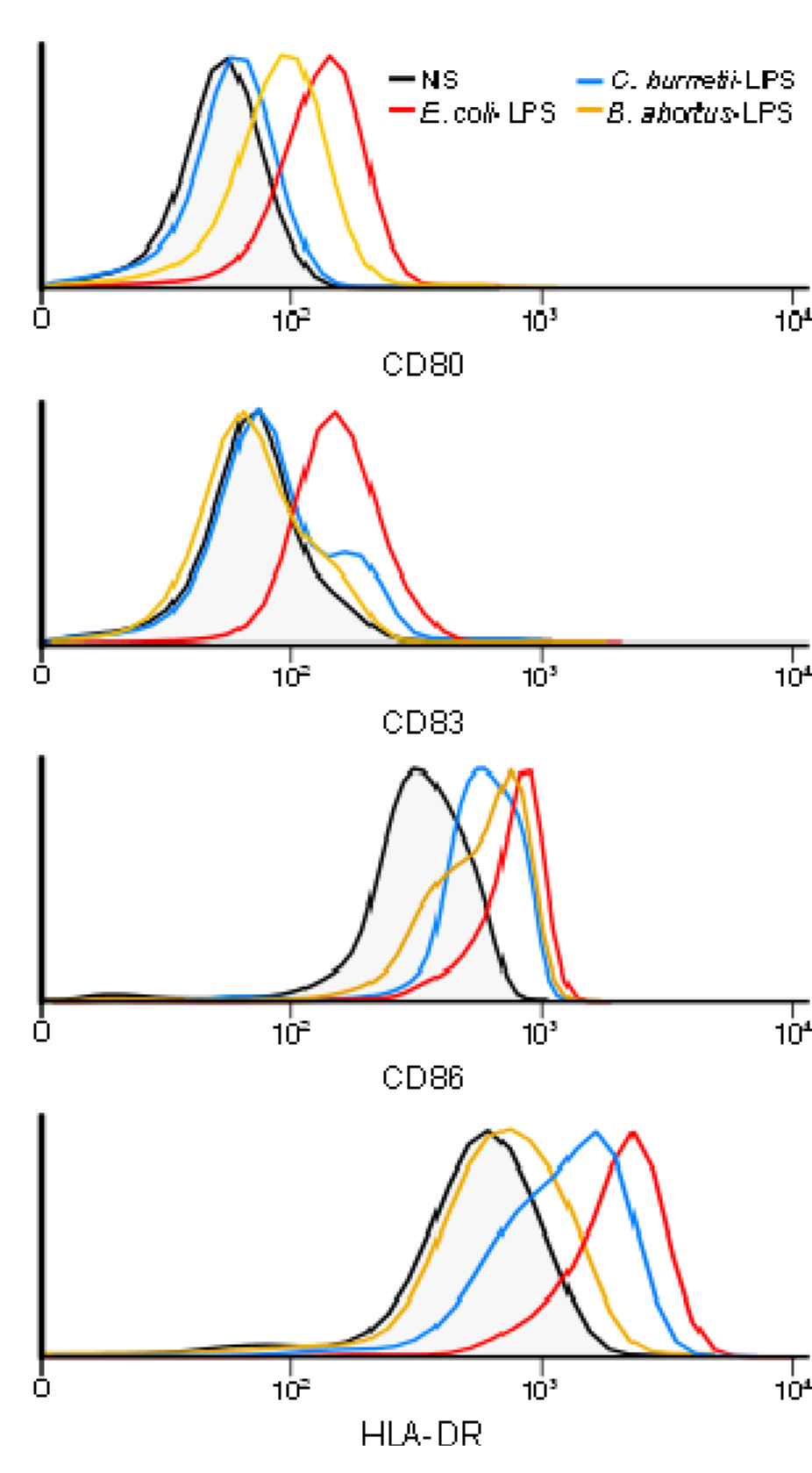

Supplement: Figure S1 — Phenotypic study of moDC maturation. moDCs were stimulated with C. burnetii-LPS, B. abortus-LPS or E. coli-LPS for 24 hours. The cells were then incubated with FITC-coupled anti-CD80, PE-coupled anti-CD83, PE-coupled anti-CD86 and FITC-coupled anti-HLA-DR Abs for 30 minutes and analyzed by flow cytometry. The curves are representative of three different experiments. (TIF) [file pone.0099420.s001.tif]
