# Supplementary material for: Intracellular Bacteria Interfere with Dendritic Cell Functions: Role of the Type I Interferon Pathway
Source: PLoS One. 2014 Jun 10;9(6):e99420. doi: 10.1371/journal.pone.0099420 (PMC4051653; doi:10.1371/journal.pone.0099420)
Supplement: Table S1 — qRT-PCR validation of genes involved in the IFN pathway. (DOC) [file pone.0099420.s002.doc]

| **Symbol** | **FC (Cb)** | | **FC (Ba)** | | **FC (Ot)** | | **FC (LPS)** | |
| --- | --- | --- | --- | --- | --- | --- | --- | --- |
|  | µarray | RT-PCR | µarray | RT-PCR | µarray | RT-PCR | µarray | RT-PCR |
| USP18 | 0.46 | 1.09 | 1.10 | 2.56 | 4.16 | 3.58 | 13.52 | 17.99 |
| HERC5 | 0.47 | 2.05 | 1.30 | 2.28 | 14.08 | 6.84 | 36.91 | 50.64 |
| Hu-IFIT5 | 1.20 | 3.15 | 1.05 | 7.19 | 4.24 | 15.06 | 6.03 | 58.61 |
| Hu-IFIT3 | 0.57 | 1.30 | 0.99 | 2.27 | 8.21 | 10.61 | 30.23 | 33.56 |
| IRF7 | 1.08 | 2.01 | 1.45 | 1.95 | 2.60 | 2.49 | 7.44 | 5.62 |
| IFIT2 | 0.80 | 1.13 | 1.43 | 2.07 | 25.5 | 10.53 | 55.45 | 25.36 |
| IFI44 | 0.45 | 1.08 | 0.97 | 1.25 | 3.63 | 2.75 | 11.52 | 8.56 |
| CXCL9 | 2.76 | 0.92 | 4.72 | 1.34 | 32.9 | 12.33 | 84.21 | 23.10 |
| IFIH1 | 1.62 | 1.73 | 1.61 | 2.54 | 4.57 | 3.44 | 7.93 | 4.30 |
| TNFSF10 | 1.18 | 0.47 | 1.95 | 0.81 | 20.6 | 10.94 | 43.7 | 31.35 |

**Table S1. qRT-PCR validation of genes involved in the IFN pathway**

moDCs were stimulated with *E. coli* LPS or bacterial pathogens for 6 hours. RNAs were extracted, and microarrays and qRT-PCR on genes involved in the IFN pathway were performed. The values of FCs obtained with both methods were compared. Ba: *B. abortus*; Cb: *C. burnetii*; Ot: *O. tsutsugamushi*.
