# Supplementary material for: Intracellular Bacteria Interfere with Dendritic Cell Functions: Role of the Type I Interferon Pathway
Source: PLoS One. 2014 Jun 10;9(6):e99420. doi: 10.1371/journal.pone.0099420 (PMC4051653; doi:10.1371/journal.pone.0099420)
Supplement: Table S2 — IL-15 and IL-12-associated genes. (DOC) [file pone.0099420.s003.doc]

**Table S2. IL-15 and IL-2-associated genes**

| **gene** | **accession number** | ***C. burnetii***  **vs. NS** | ***B. abortus***  **vs. NS** | ***O. tsutsugamushi***  **vs. NS** | **LPS vs. NS** |
| --- | --- | --- | --- | --- | --- |
| IL15 | NM_172174 | 5.94 | 0.79 | 3.44 | 10.27 |
| IL15RA | NM_172200 | 29.25 | 1.27 | 3.31 | 67.68 |
| IL2RA | NM_000417 | 62.84 | 7.72 | 1.33 | 52.77 |
| IL2RG | NM_000206 | 1.78 | 2.51 | 0.67 | 1.51 |
| IL2RB | NM_000878 | 2.00 | 0.53 | 1.13 | 1.87 |
| IL2 | NM_000586 | 1.51 | 0.92 | 0.35 | 3.11 |

moDCs were stimulated with bacterial pathogens or E. coli LPS for 6 hours. RNAs were extracted, and microarrays were performed. The level of expression of genes associated with IL-15 and IL-2 is calculated relative to unstimulated condition (NS). IL2RA: alpha chain of IL2R or CD25; IL2RB: beta chain of IL2R or CD122; IL2RG: gamma chain of IL2R or CD132.
