# Supplementary material for: Intracellular Bacteria Interfere with Dendritic Cell Functions: Role of the Type I Interferon Pathway
Source: PLoS One. 2014 Jun 10;9(6):e99420. doi: 10.1371/journal.pone.0099420 (PMC4051653; doi:10.1371/journal.pone.0099420)
Supplement: Results S1 — Pathway analysis of transcriptional modulation of moDC by intracellular bacteria. (PDF) [file pone.0099420.s004.pdf]

Intracellular bacteria interfere with dendritic cell function: Role of type I Interferon pathway

L. Gorvel, J. Textoris, R. Banchereau, A. Ben Amara, W. Tantibhedhyangkul, K. von Bargen, M.B. Ka, C. Capo, E. Ghigo, J.P. Gorvel and J.L. Mege

Supplementary Results S1: Pathway Analysis of Transcriptional Modulation of moDC by Intracellular Bacteria

Based on the functional analysis of the genes modulated by the intracellular bacteria or LPS in moDC, we extracted the identified KEGG pathways and color coded the nodes according to the modulation of gene expression after each stimulation.

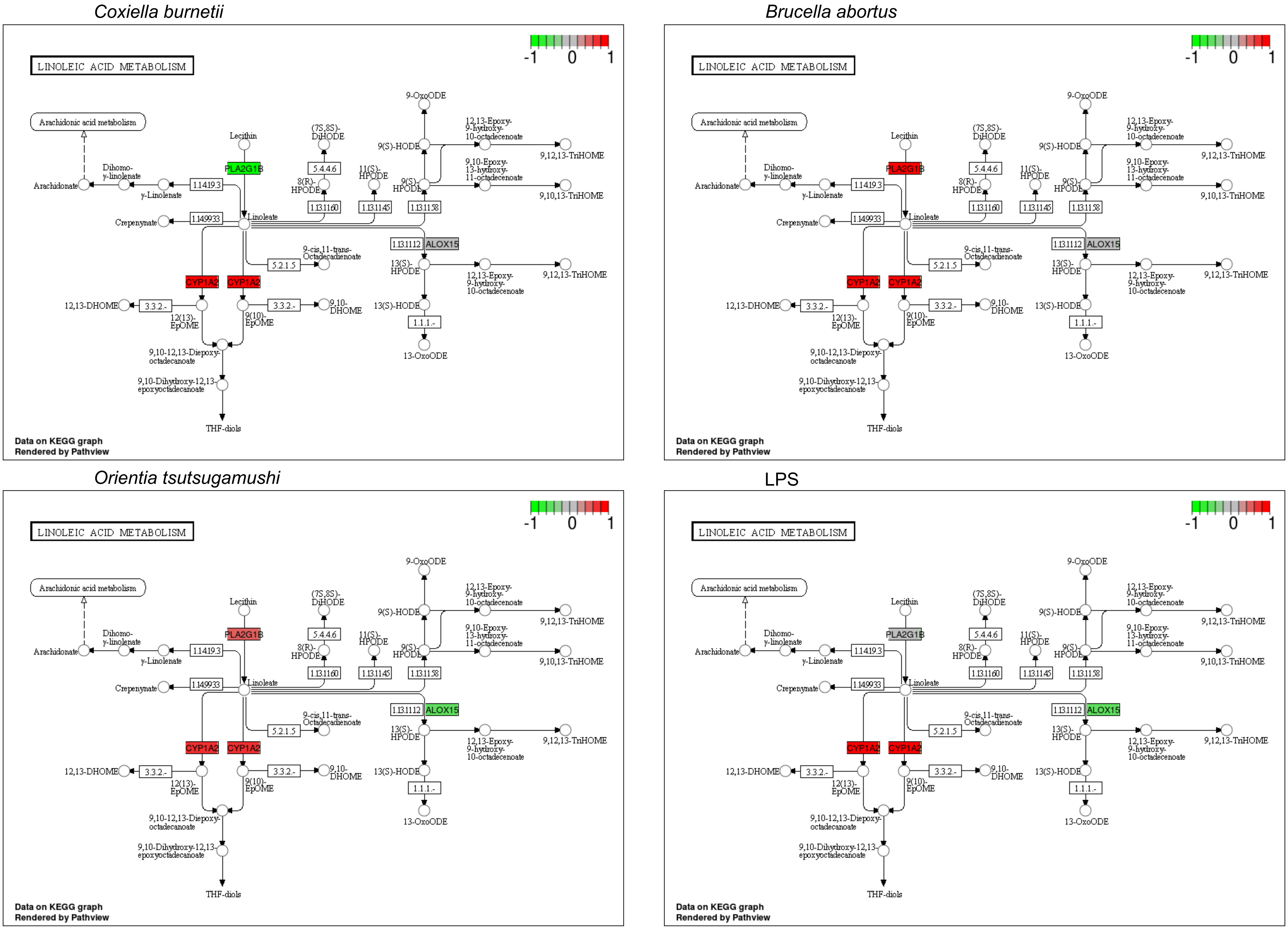
Coxiella burnetii
Brucella abortus
Orientia tsutsugamushi
LPS
LINOLEIC ACID METABOLISM
-1 0 1
Arachidonic acid metabolism
Arachidonate
Dihomo-γ-linolenate
γ-Linolenate
1.14.19.3
Lecithin
PLA2G1B
Linoleate
Crepenynate
1.14.99.33
(7S,8S)-DiHODE
5.4.4.6
8(R)-HPODE
1.13.11.60
11(S)-HPODE
1.13.11.45
9(S)-HPODE
1.13.11.58
9-OxoODE
9(S)-HODE
12,13-Epoxy-9-hydroxy-10-octadecenoate
9,12,13-TriHOME
9,10-Epoxy-13-hydroxy-11-octadecenoate
9,10,13-TriHOME
1.13.11.12
ALOX15
9-cis,11-trans-Octadecadienoate
5.2.1.5
13(S)-HPODE
13(S)-HODE
1.1.1.-
13-OxoODE
CYP1A2
12,13-DHOME
3.3.2.-
12(13)-EpOME
9(10)-EpOME
9,10-DHOME
9,10-12,13-Diepoxy-octadecanoate
9,10-Dihydroxy-12,13-epoxyoctadecanoate
THF-diols
Data on KEGG graph
Rendered by Pathview

Coxiella burnetii

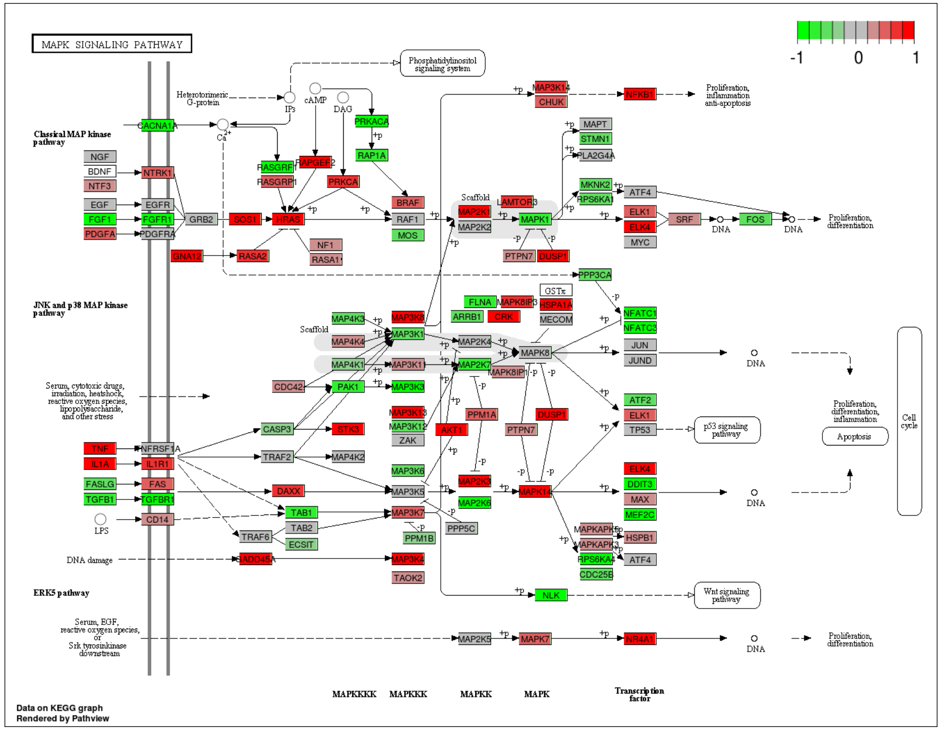

Brucella abortus

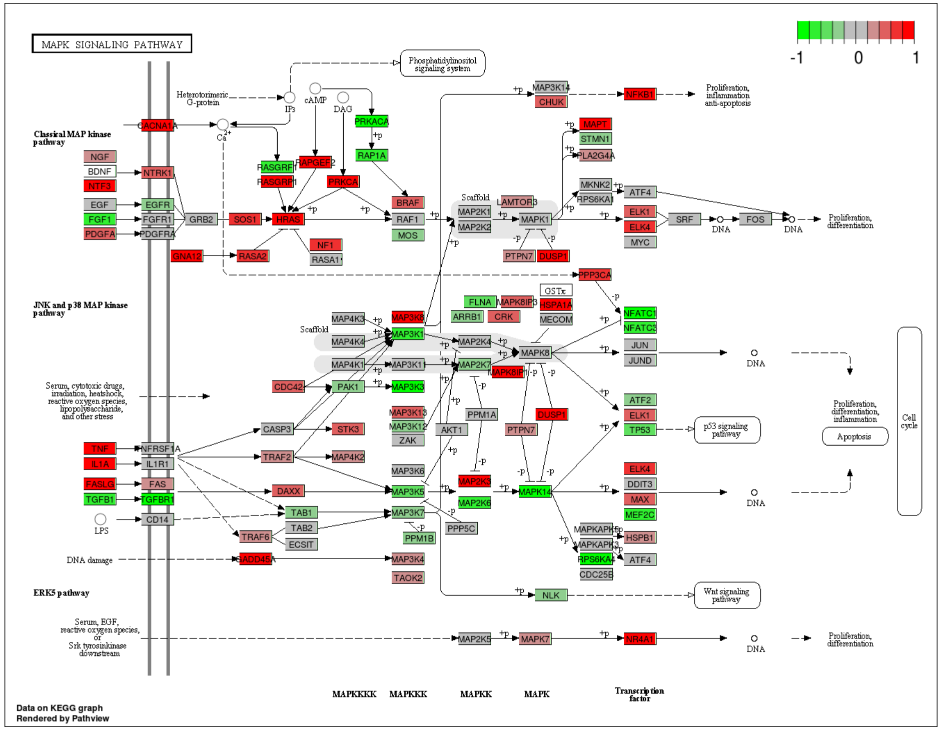

Orientia tsutsugamushi

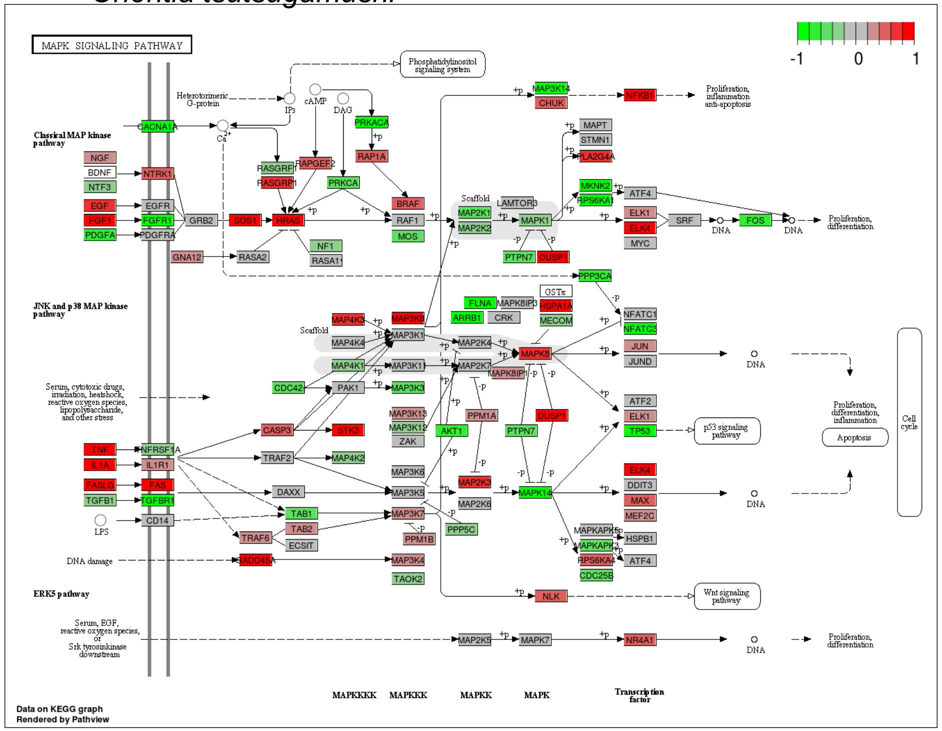

LPS

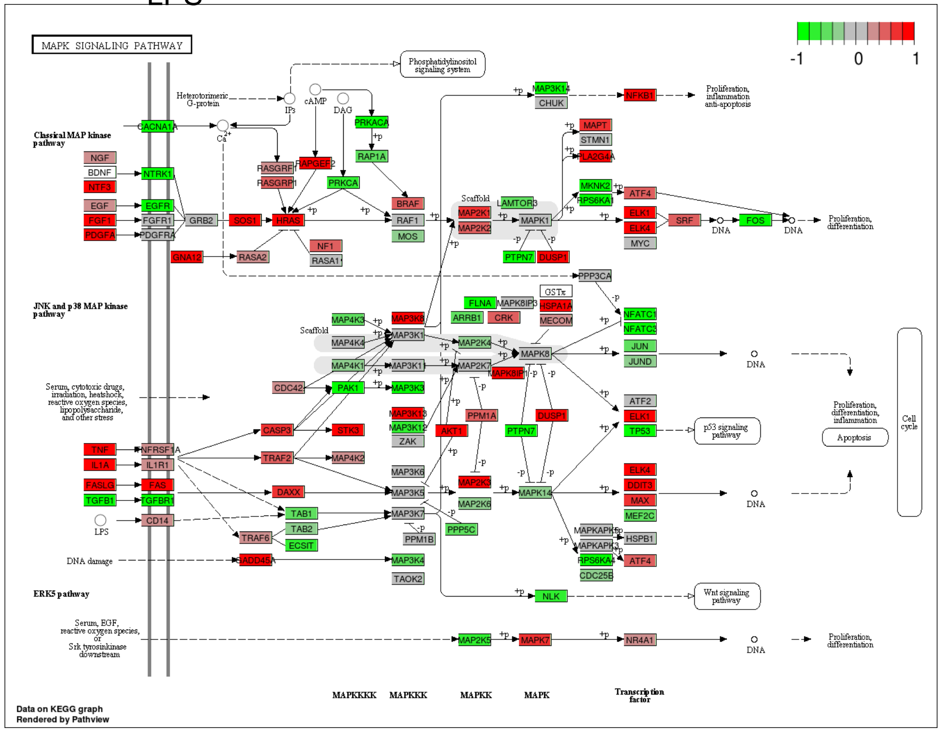

Coxiella burnetii

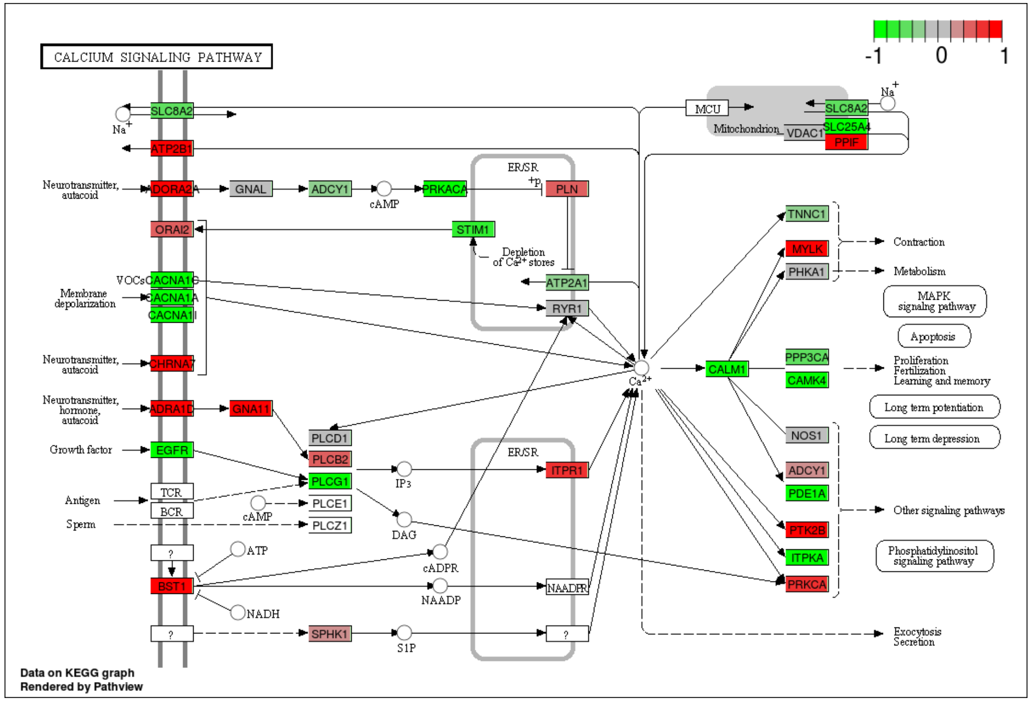

Brucella abortus

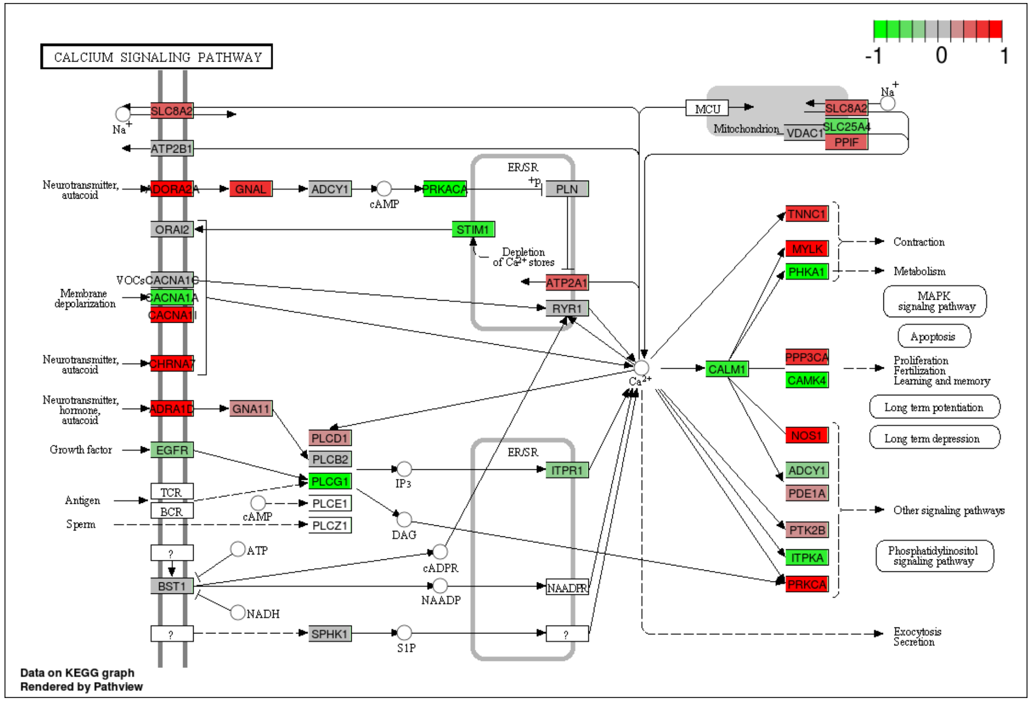

Orientia tsutsugamushi

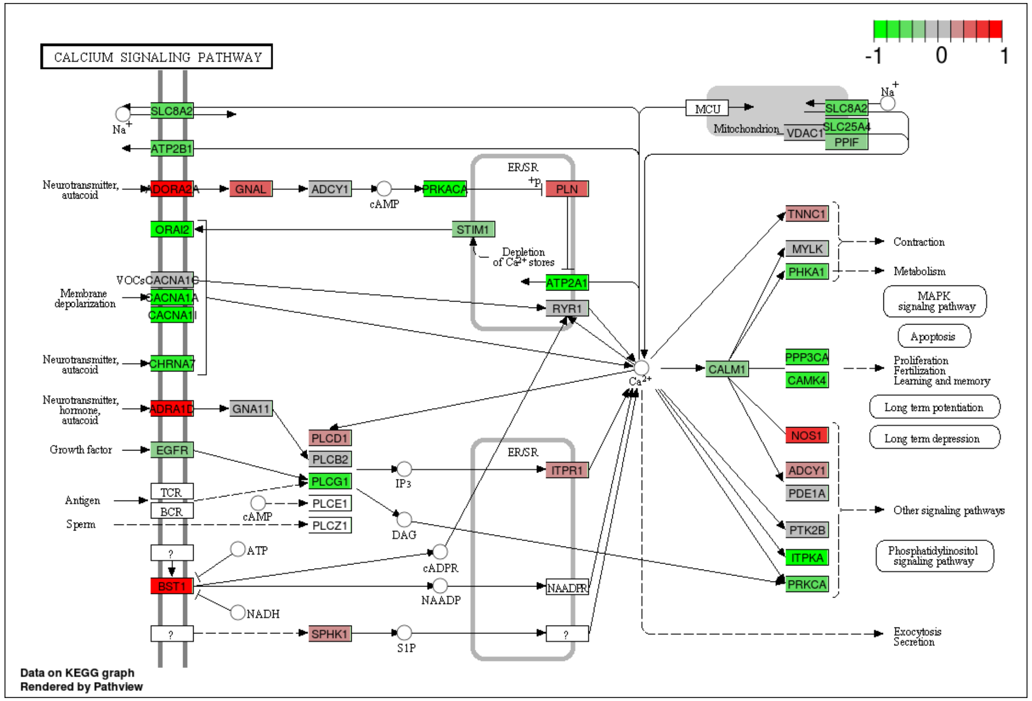

LPS

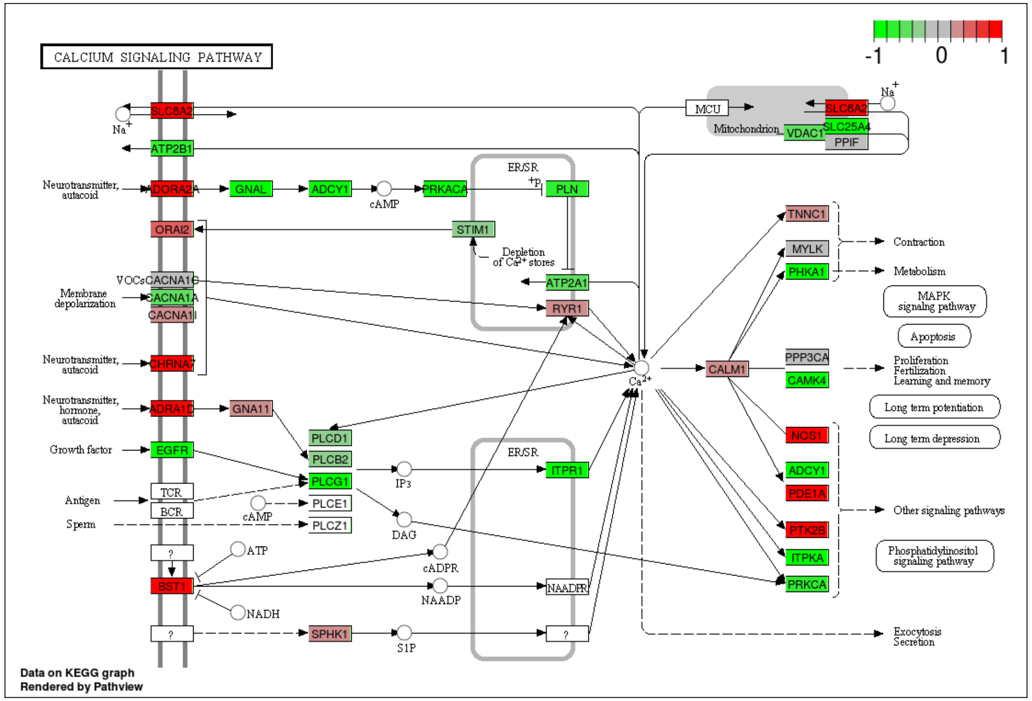

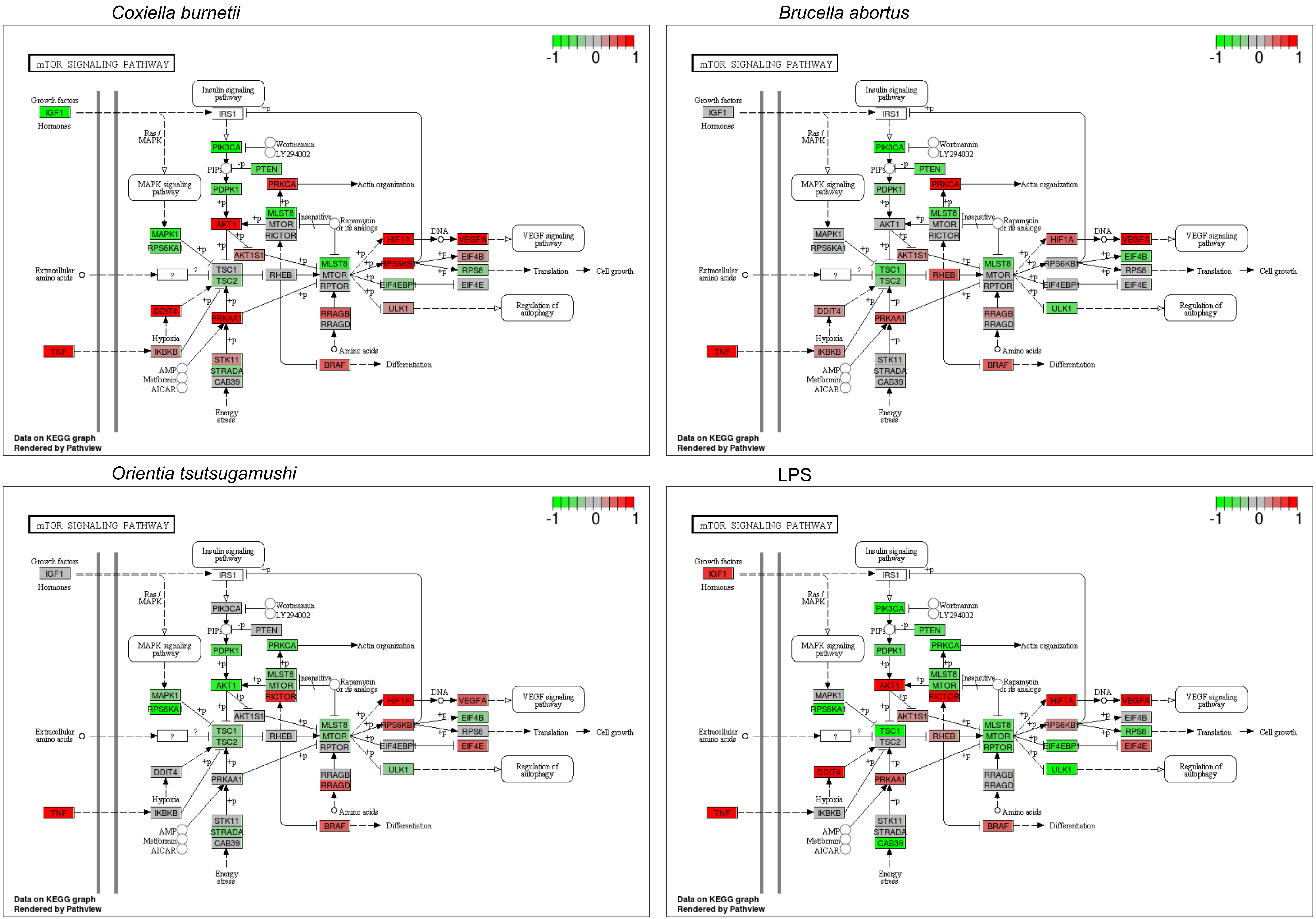
Coxiella burnetii
Brucella abortus
Orientia tsutsugamushi
LPS
-1
0
1
mTOR SIGNALING PATHWAY
Growth factors
IGF1
Hormones
Insulin signaling pathway
IRS1
Ras / MAPK
PIK3CA
Wortmannin
LY294002
PIP3
PTEN
MAPK signaling pathway
PDPK1
PRKCA
Actin organization
MLST8
MTOR
RICTOR
AKT1
Insensitive
Rapamycin or its analogs
MAPK1
RPS6KA1
AKT1S1
HIF1A
DNA
VEGFA
VEGF signaling pathway
EIF4B
RPS6KB
RPS6
Translation
Cell growth
Extracellular amino acids
?
TSC1
TSC2
RHEB
RPTOR
EIF4EBP
EIF4E
ULK1
Regulation of autophagy
DDIT4
PRKAA1
RRAGB
RRAGD
Hypoxia
TNF
IKBKB
Amino acids
BRAF
Differentiation
AMP
Metformin
AICAR
STK11
STRADA
CAB39
Energy stress
Data on KEGG graph
Rendered by Pathview

Coxiella burnetii

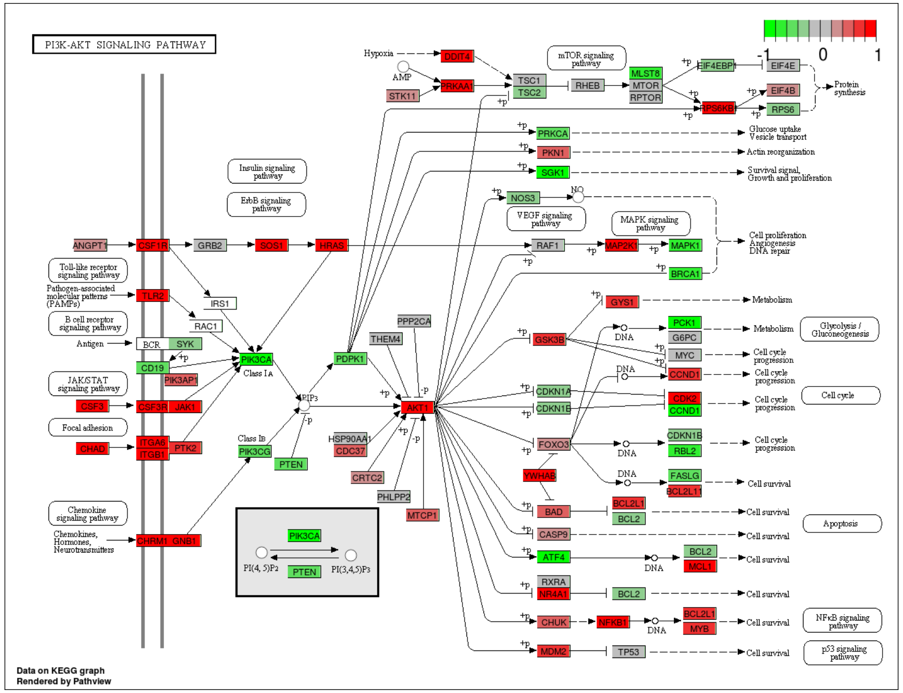

Brucella abortus

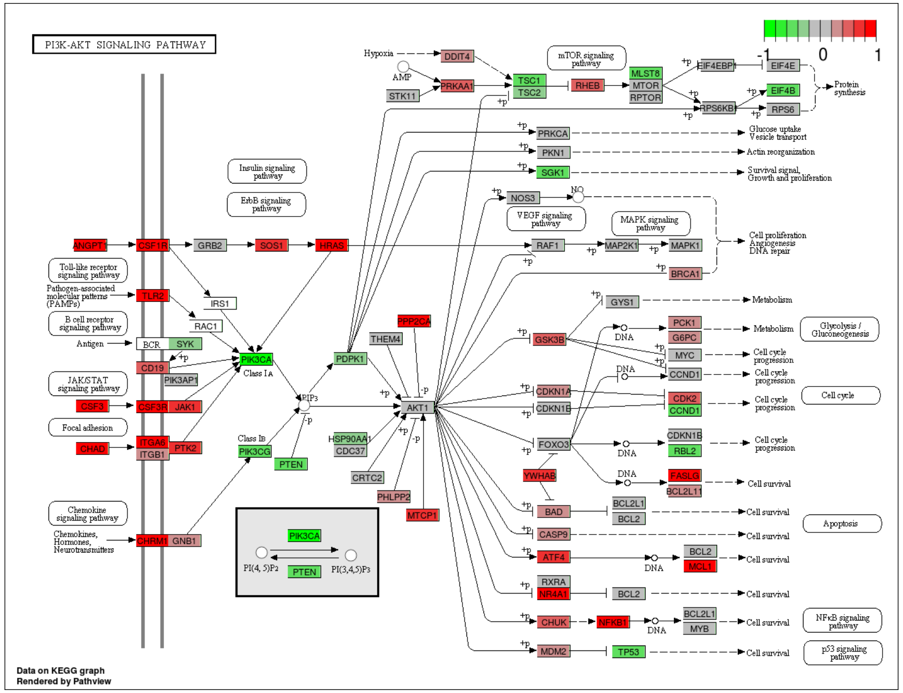

Orientia tsutsugamushi

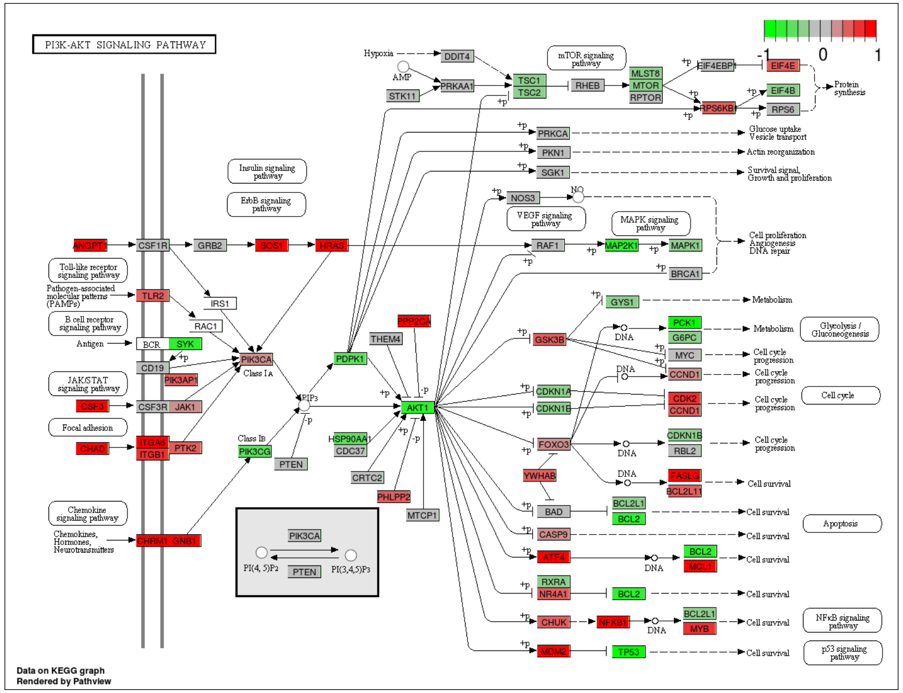

LPS

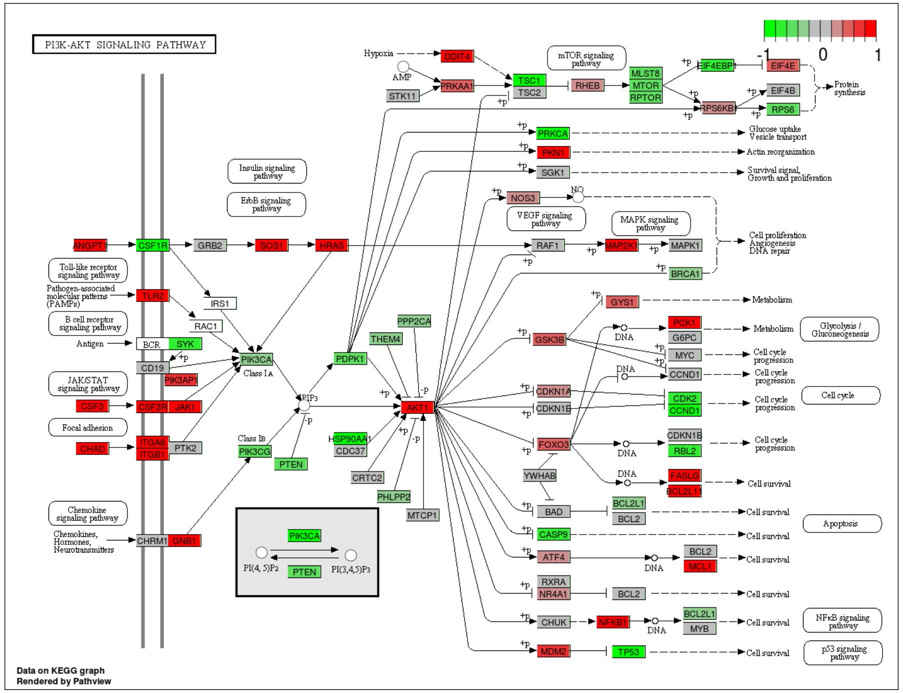

Coxiella burnetii

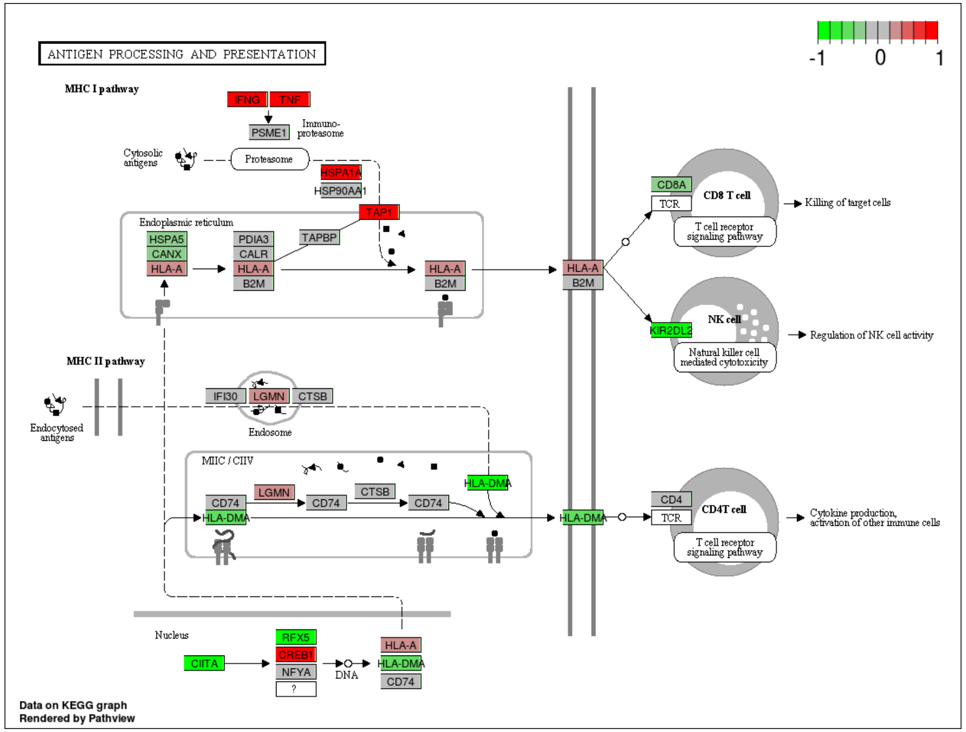

Brucella abortus

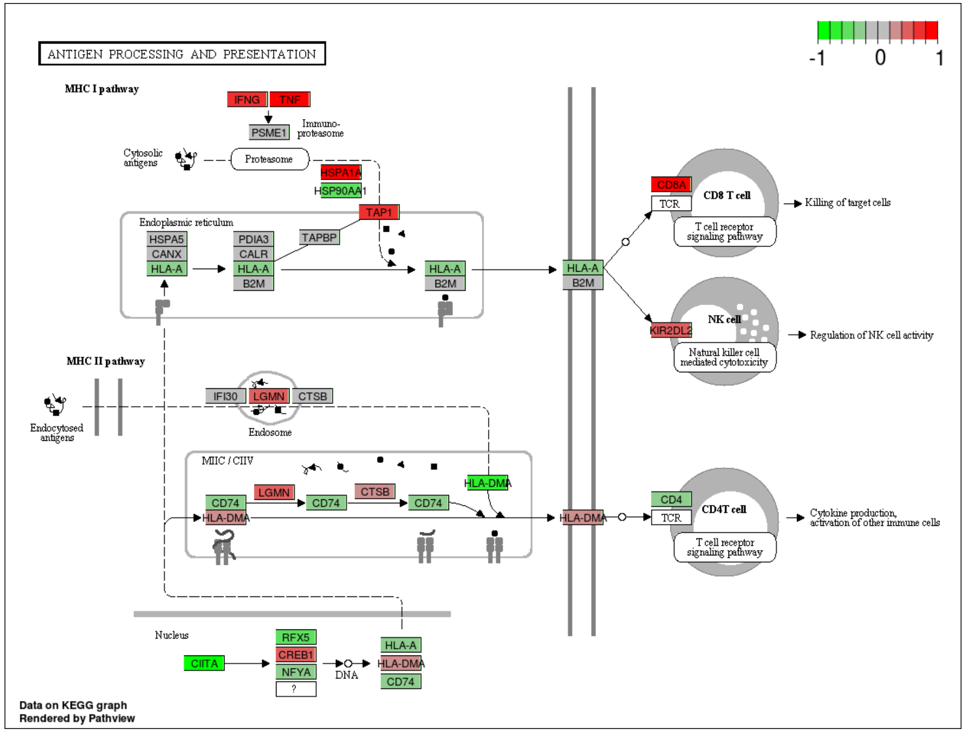

Orientia tsutsugamushi

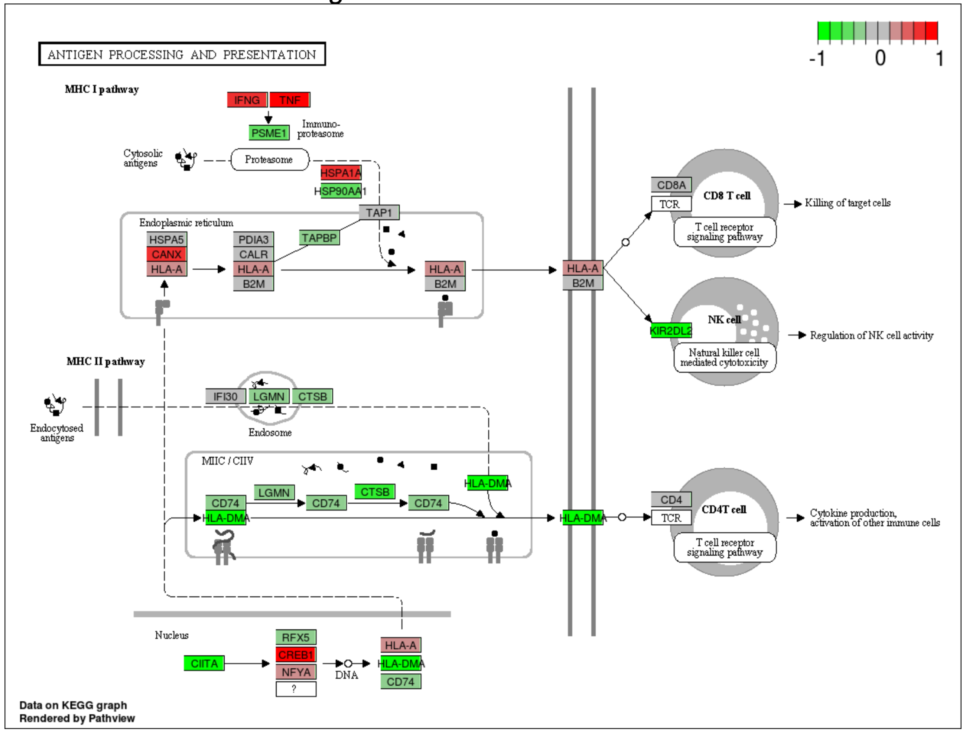

LPS

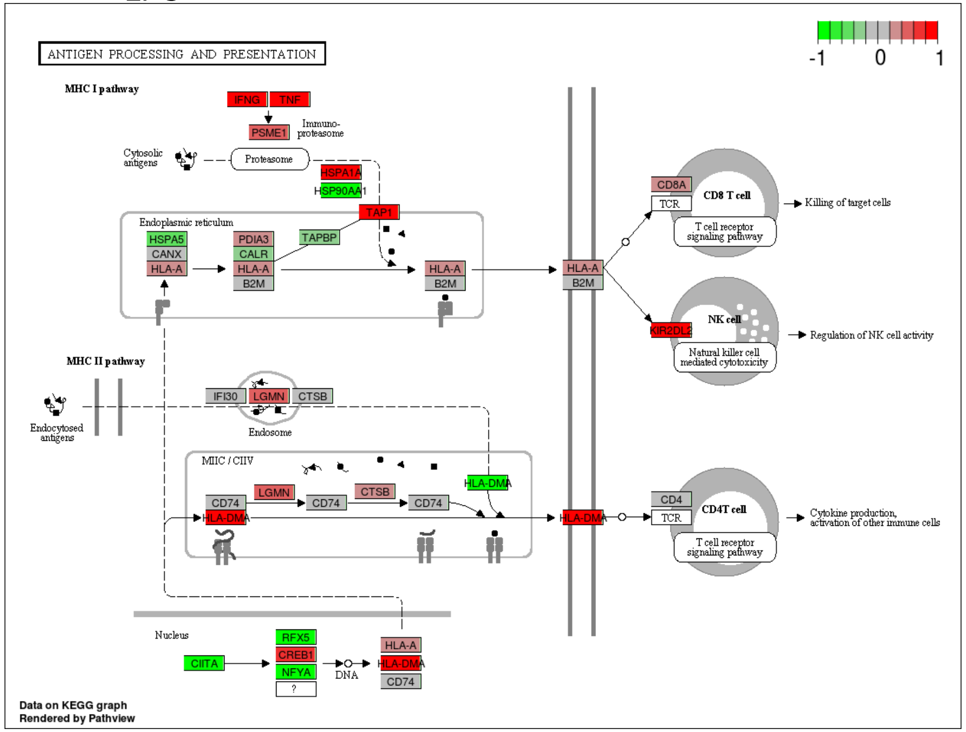

Coxiella burnetii

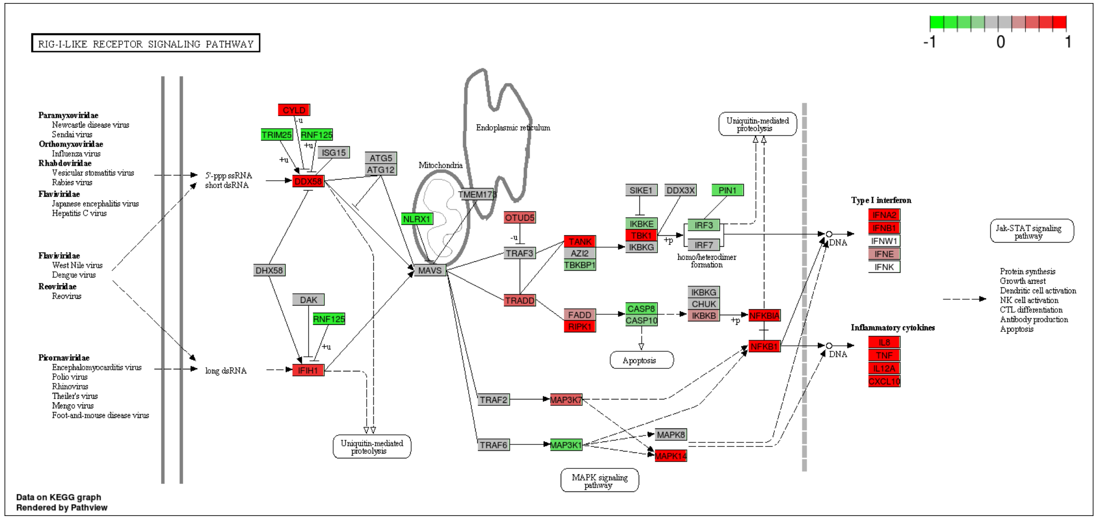

Brucella abortus

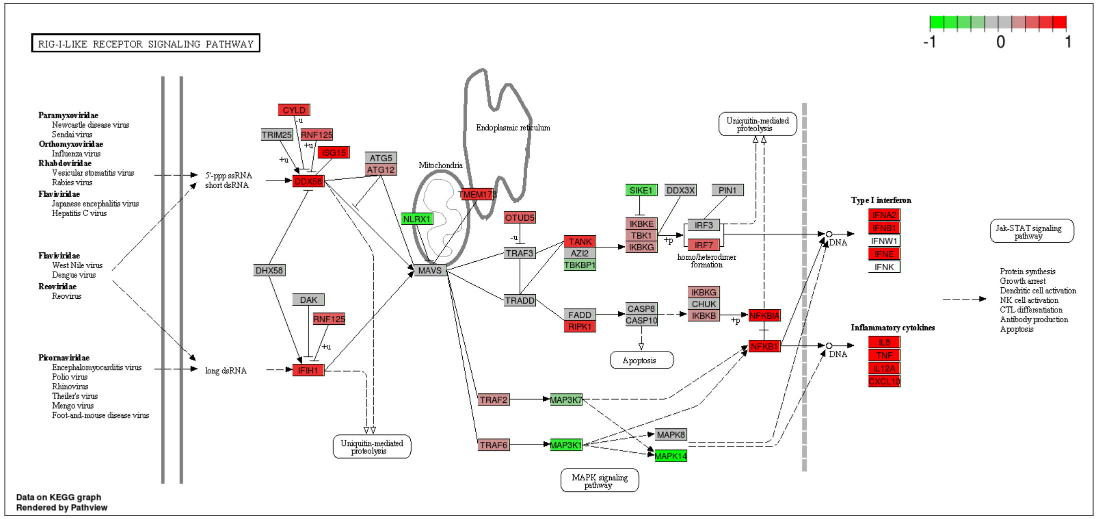

Orientia tsutsugamushi

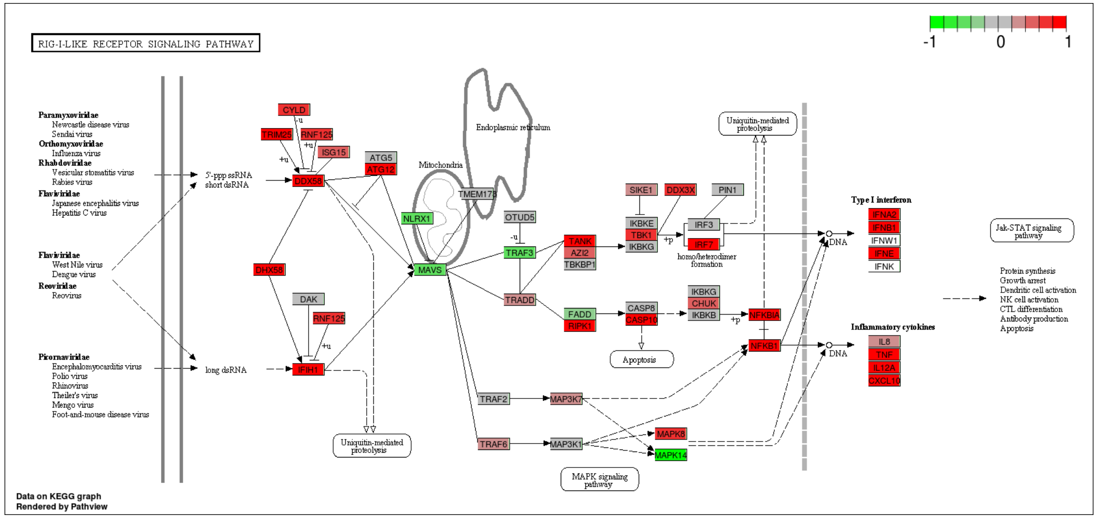

LPS

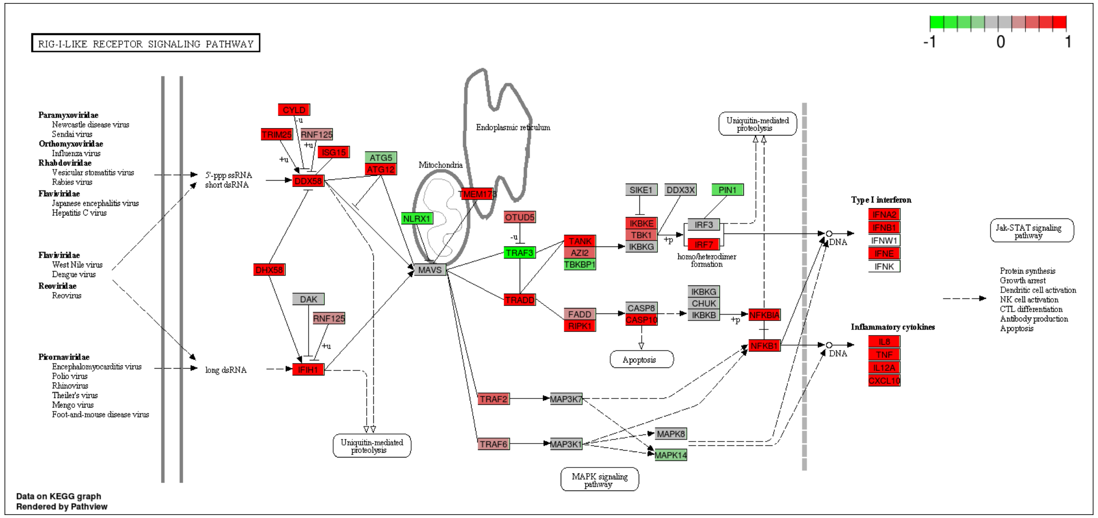

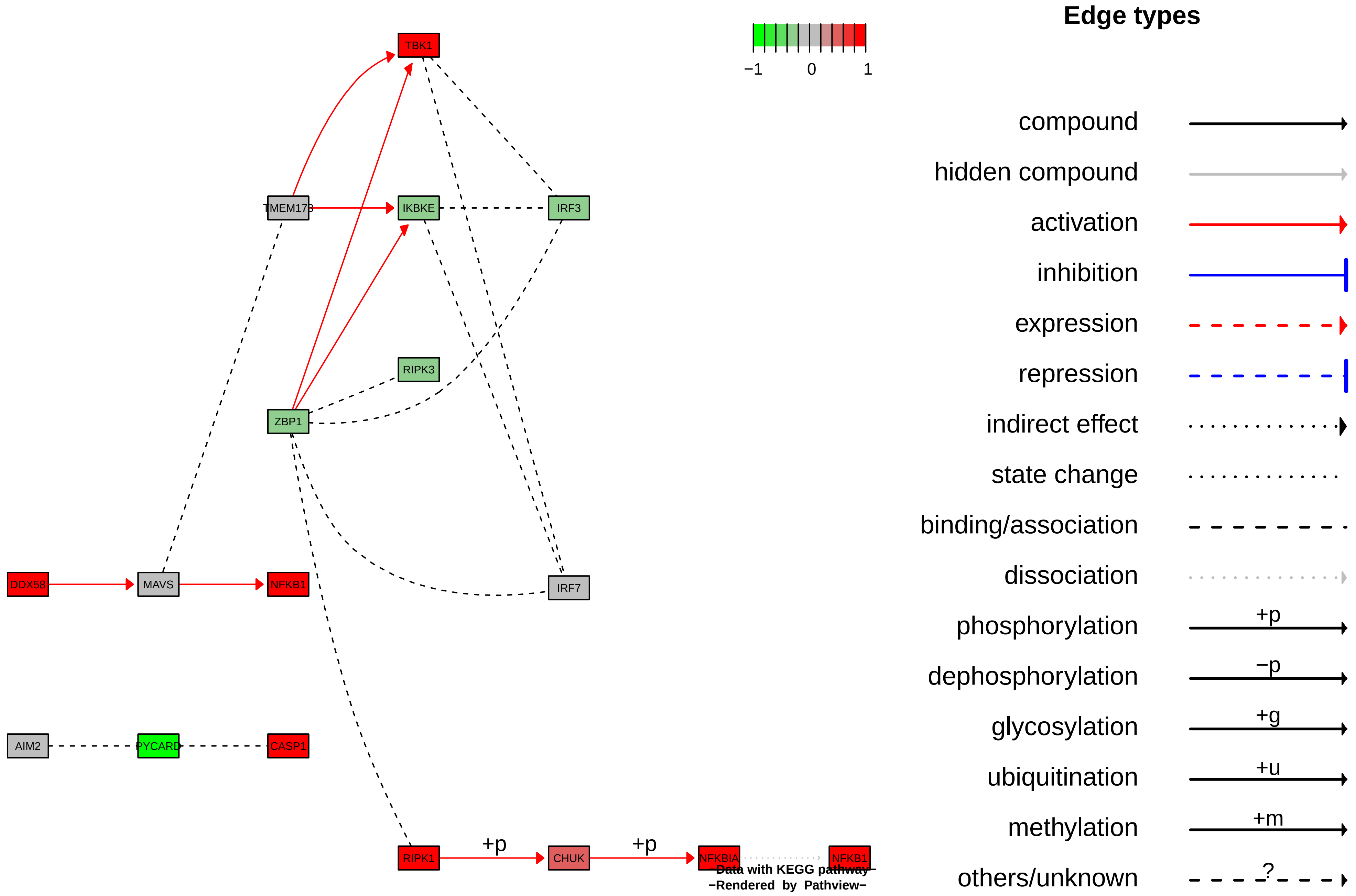

TBK1
TMEM173
IKBKE
IRF3
RIPK3
ZBP1
DDX58
MAVS
NFKB1
IRF7
AIM2
PYCARD
CASP1
RIPK1
+p
CHUK
+p
NFKBIA
NFKB1
−1
0
1
Edge types
compound
hidden compound
activation
inhibition
expression
repression
indirect effect
state change
binding/association
dissociation
phosphorylation
+p
dephosphorylation
−p
glycosylation
+g
ubiquitination
+u
methylation
+m
others/unknown
?
–Data with KEGG pathway–
–Rendered by Pathview–

Coxiella burnetii

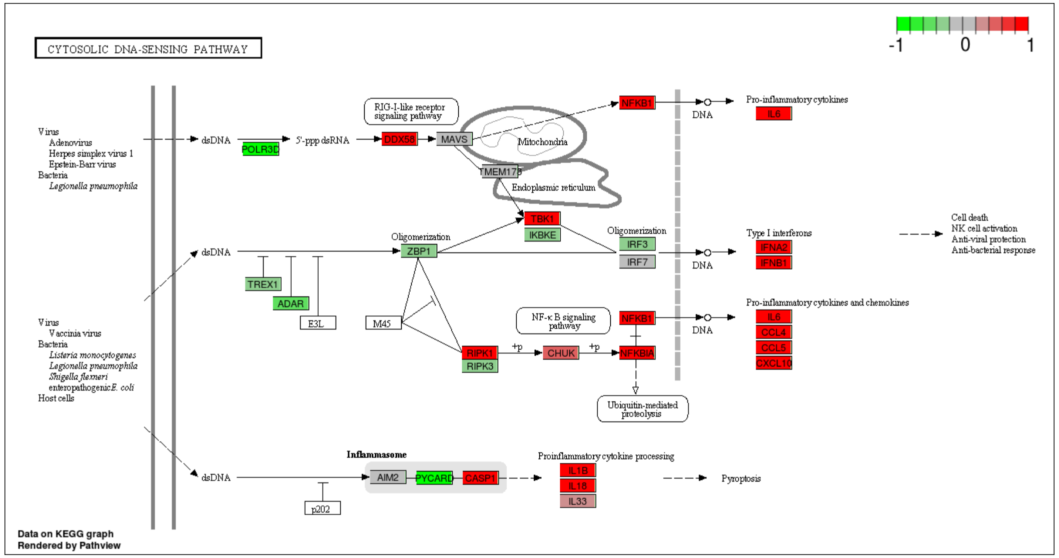

Brucella abortus

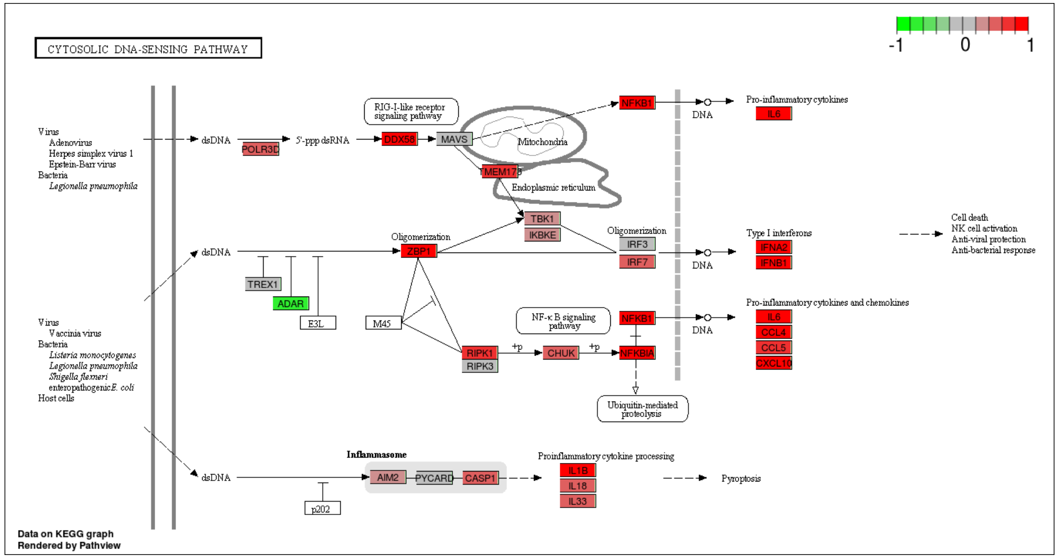

Orientia tsutsugamushi

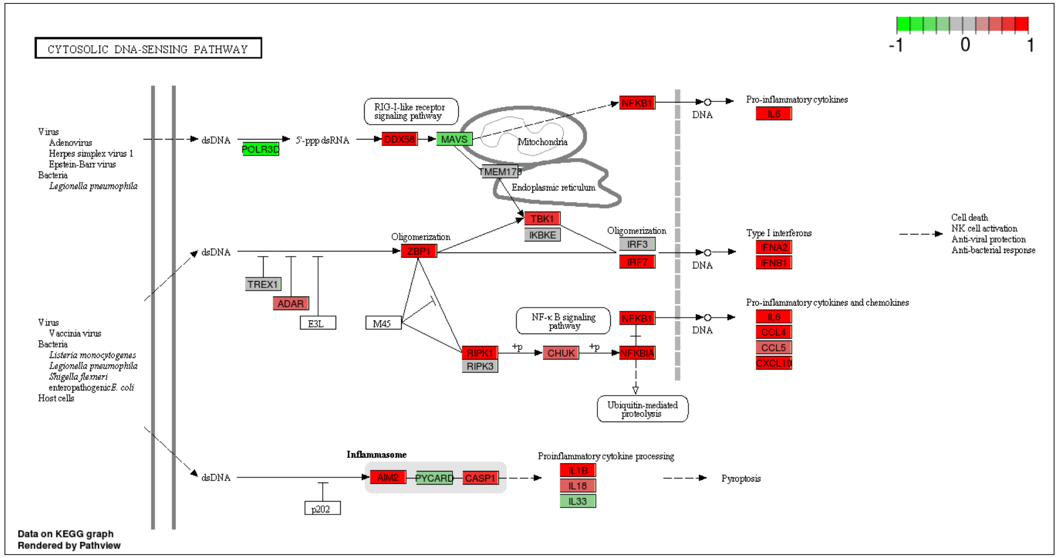

LPS

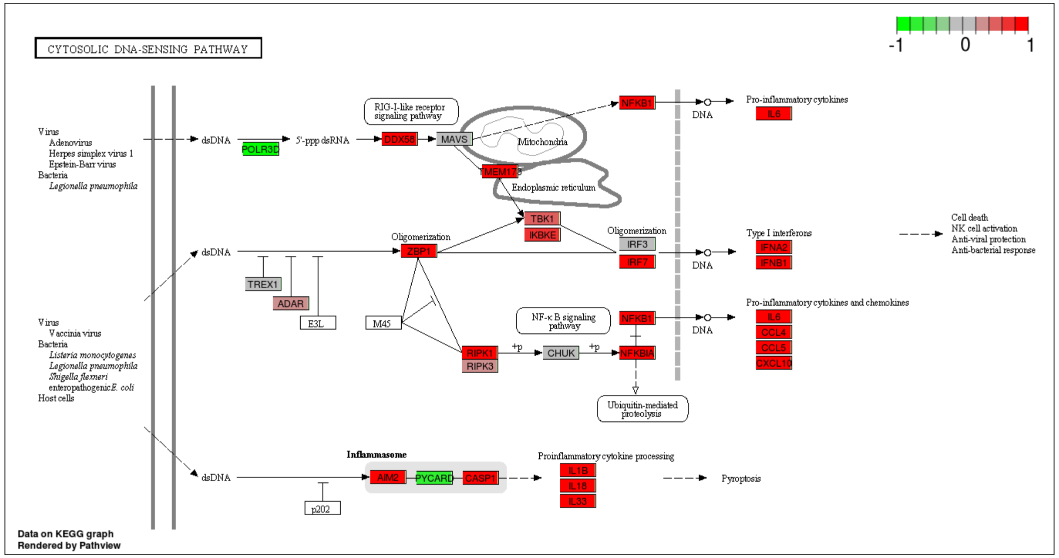

Coxiella burnetii

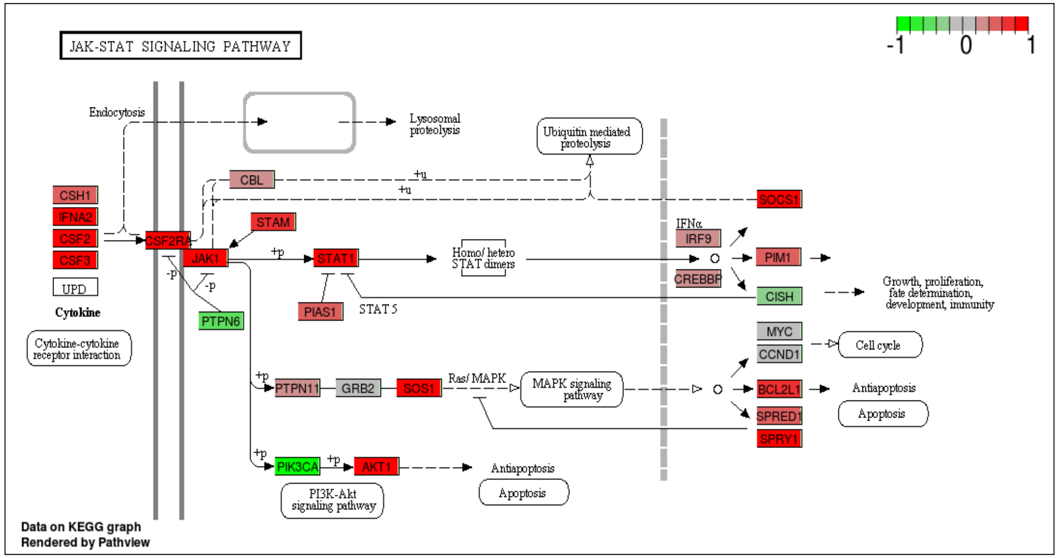

Brucella abortus

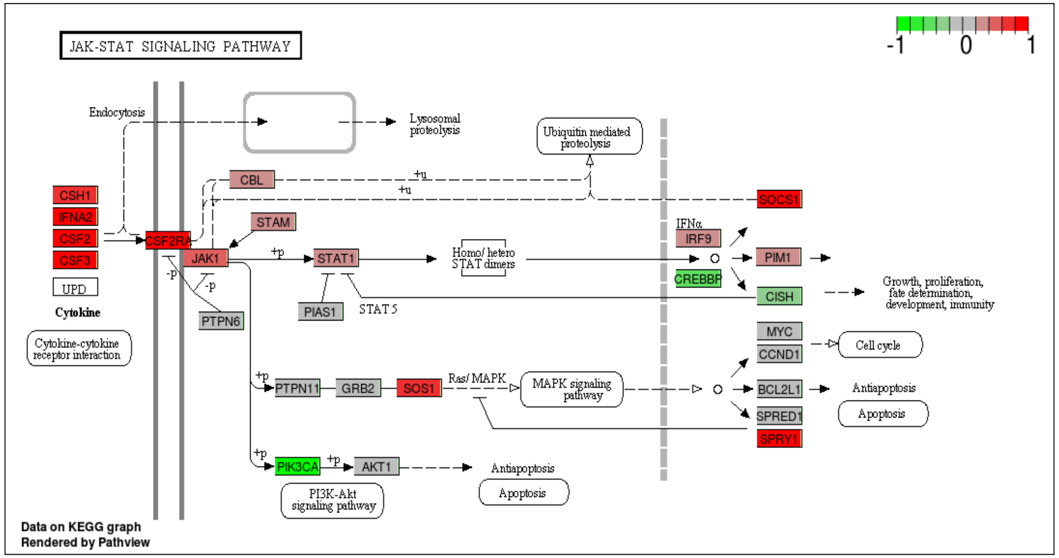

Orientia tsutsugamushi

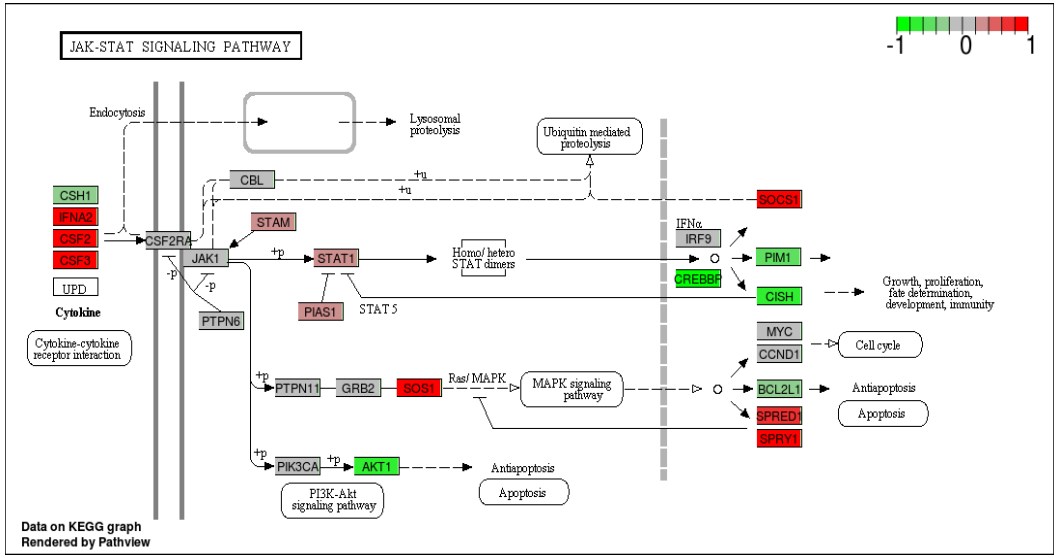

LPS

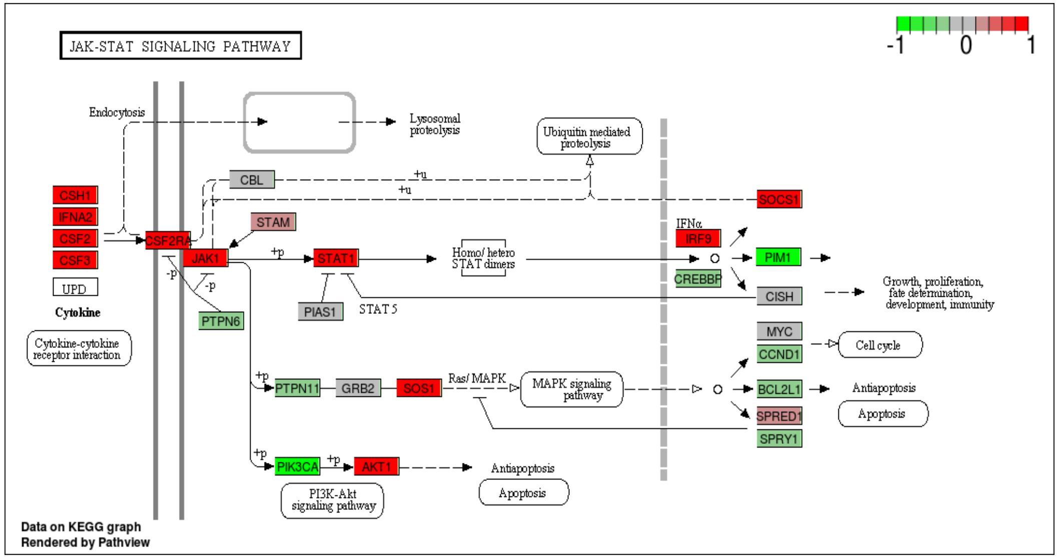

Coxiella burnetii

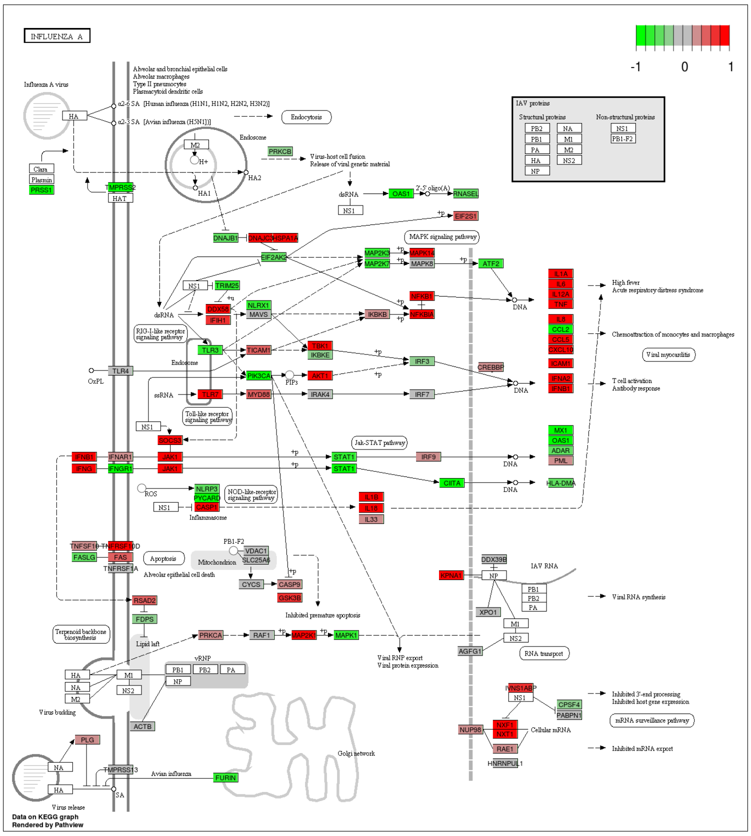

Brucella abortus

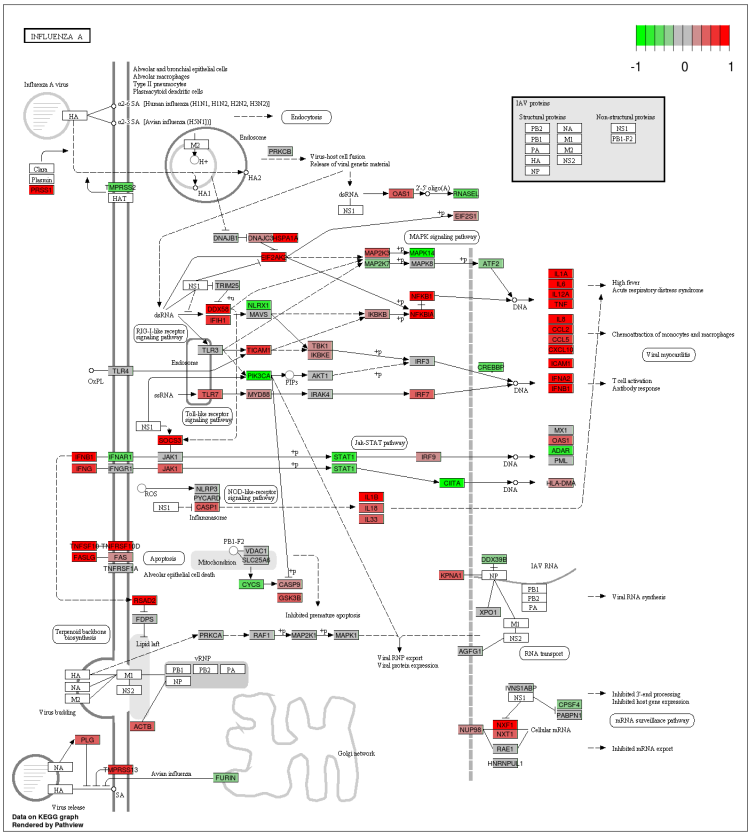

Orientia tsutsugamushi

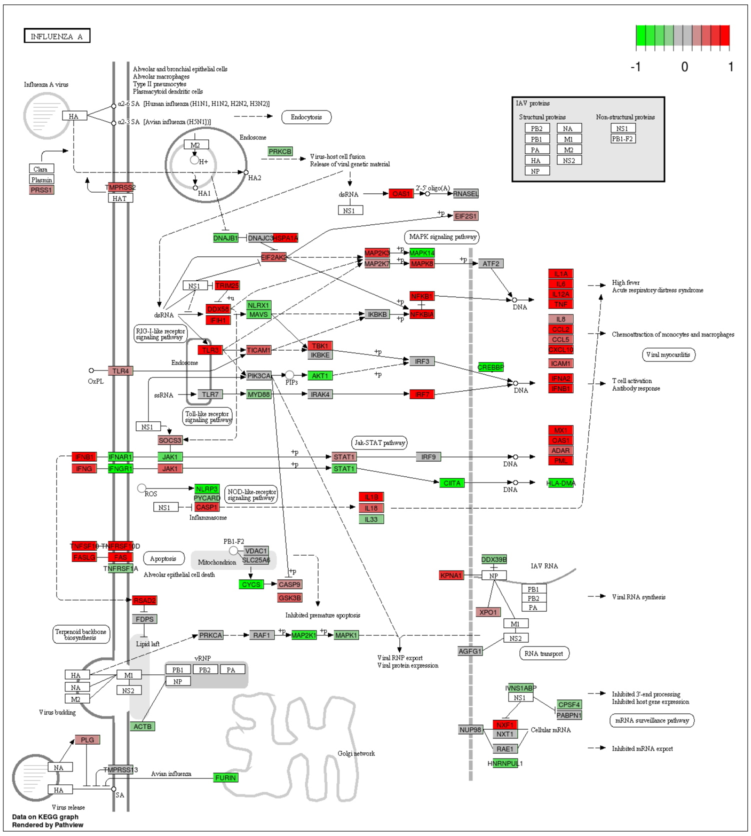

LPS

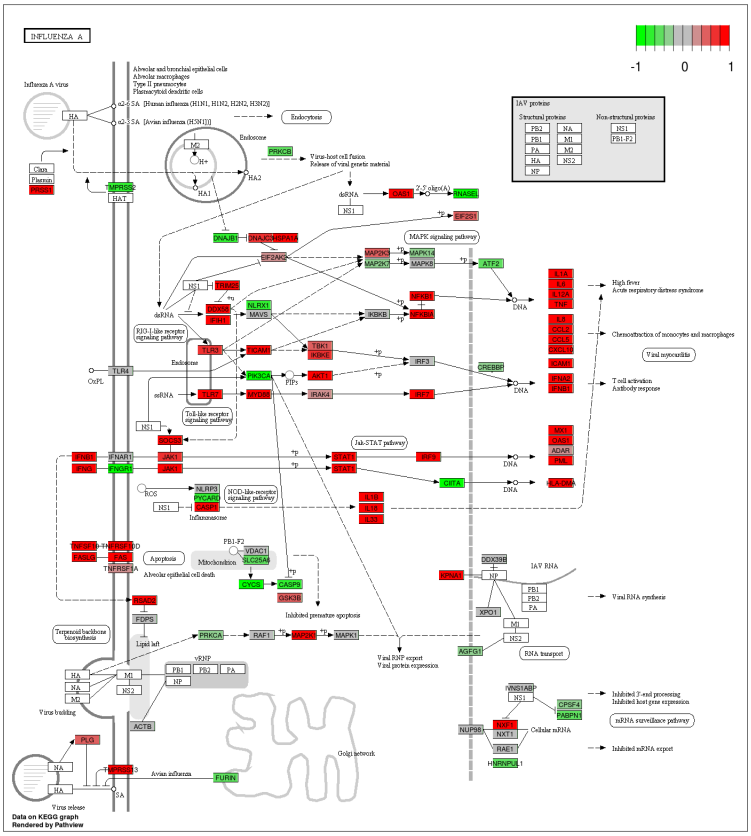

Coxiella burnetii

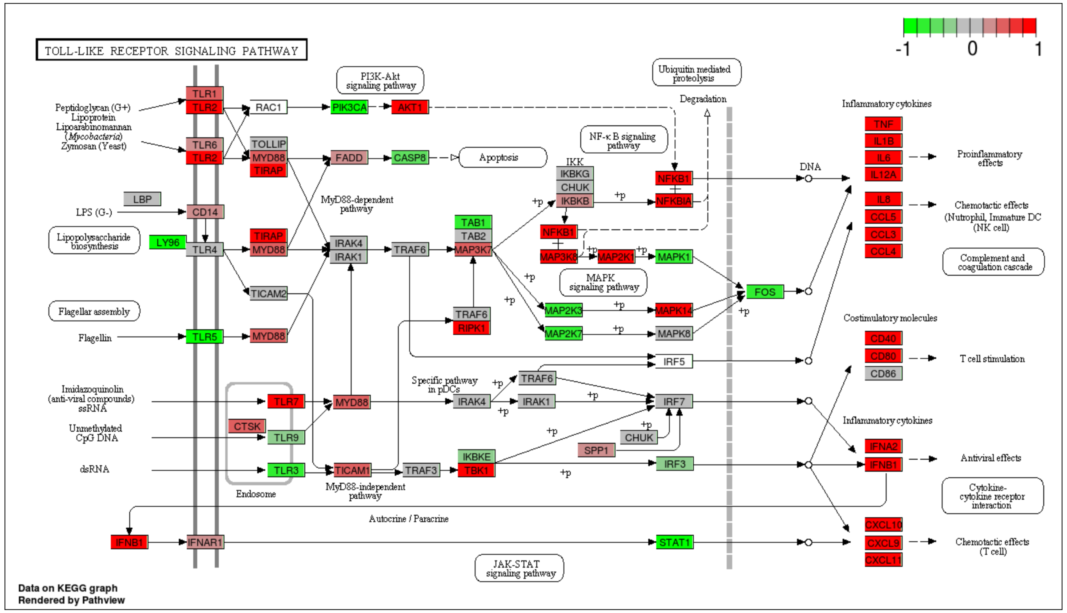

Brucella abortus

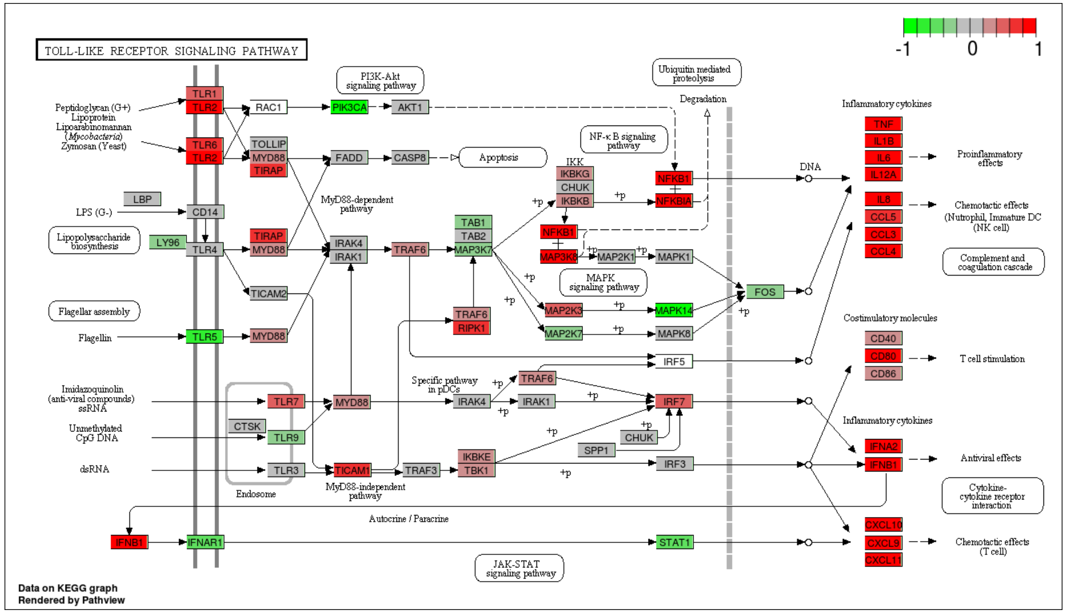

Orientia tsutsugamushi

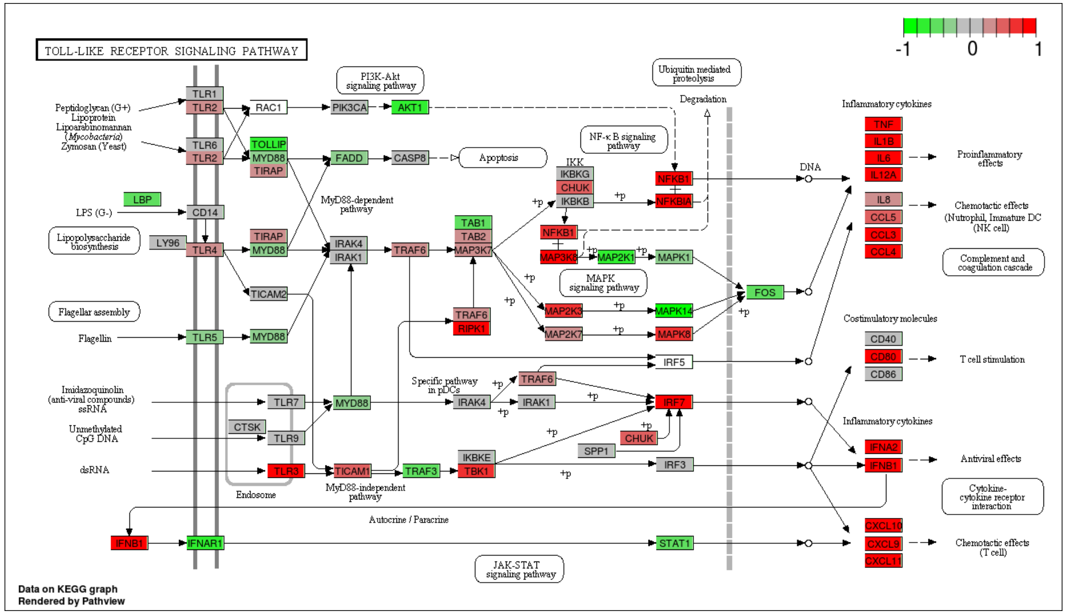

LPS

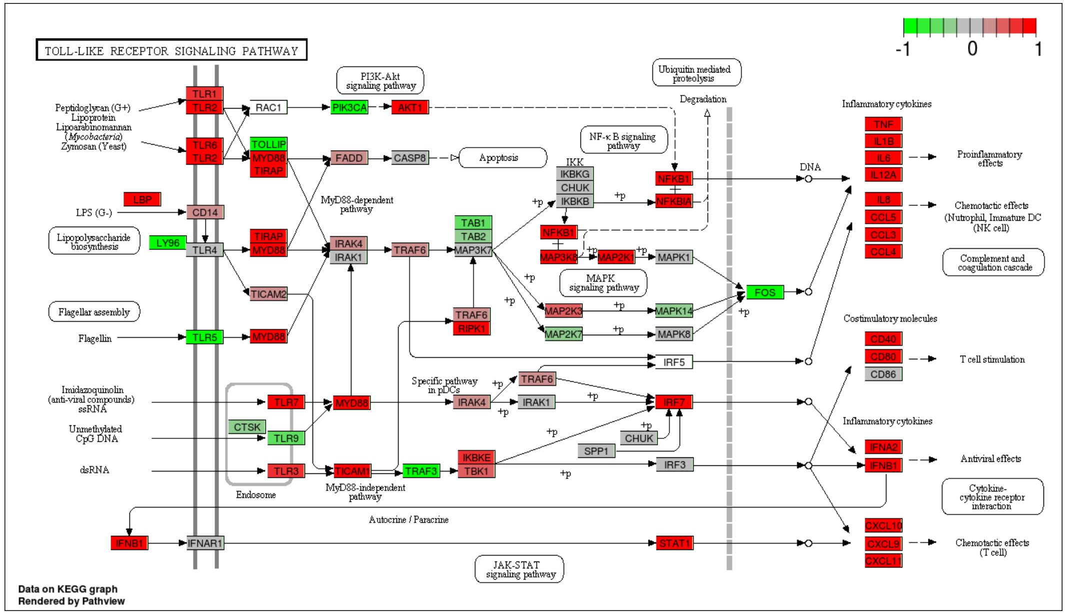

Coxiella burnetii

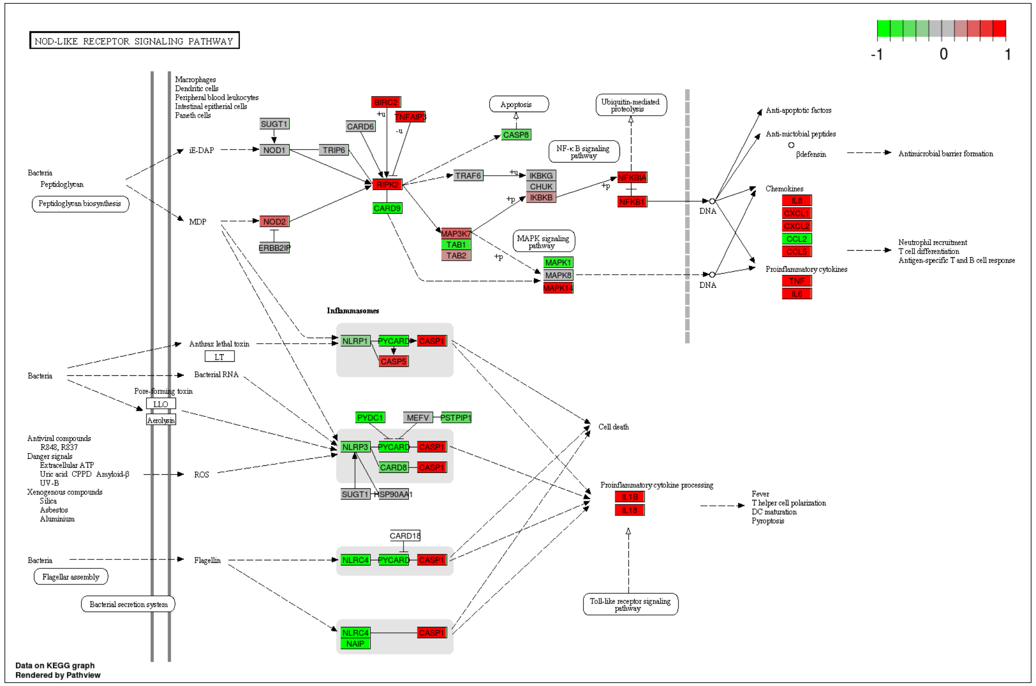

Brucella abortus

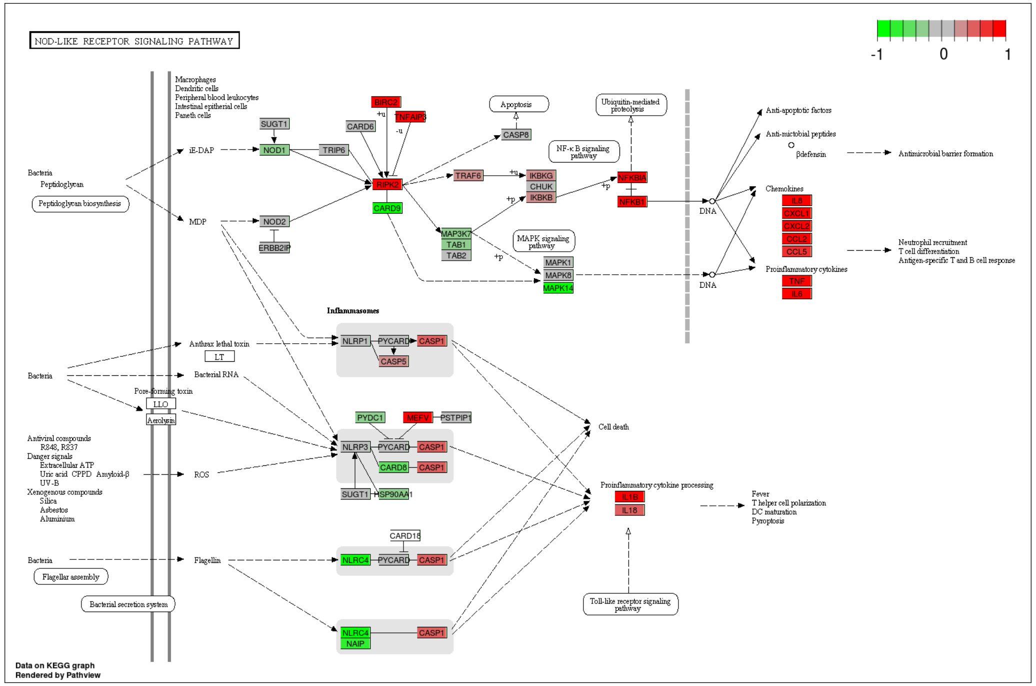

Orientia tsutsugamushi

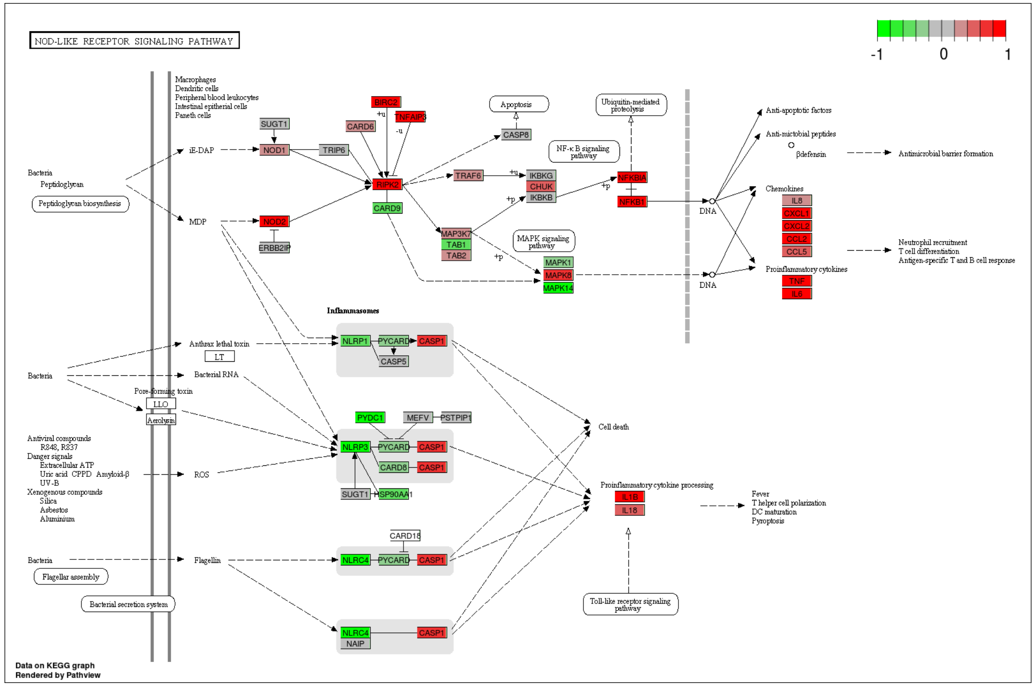

LPS

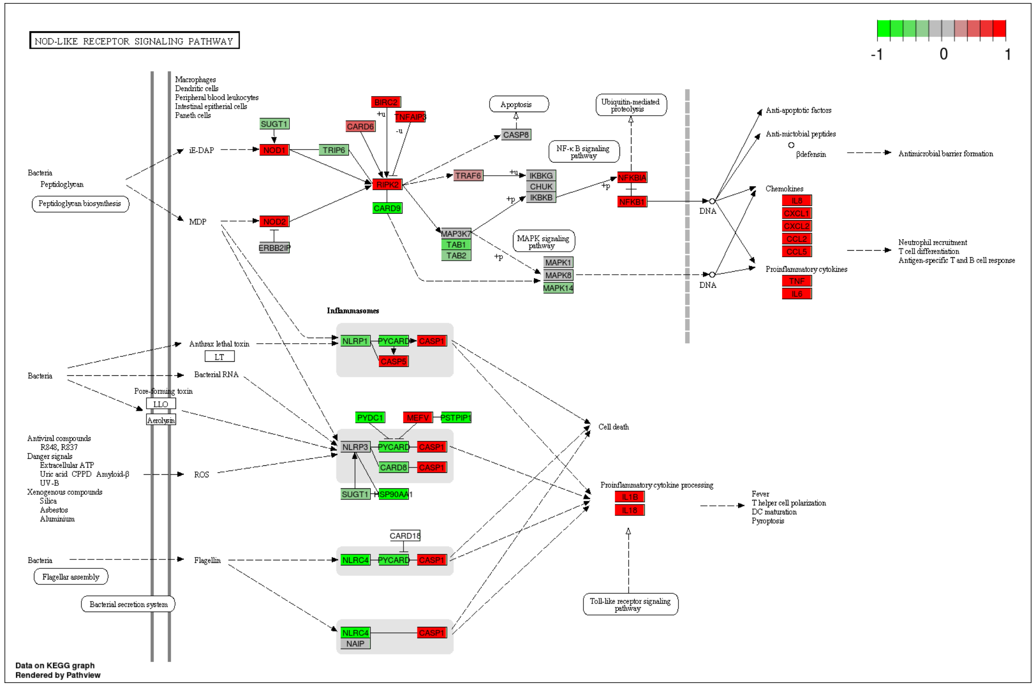

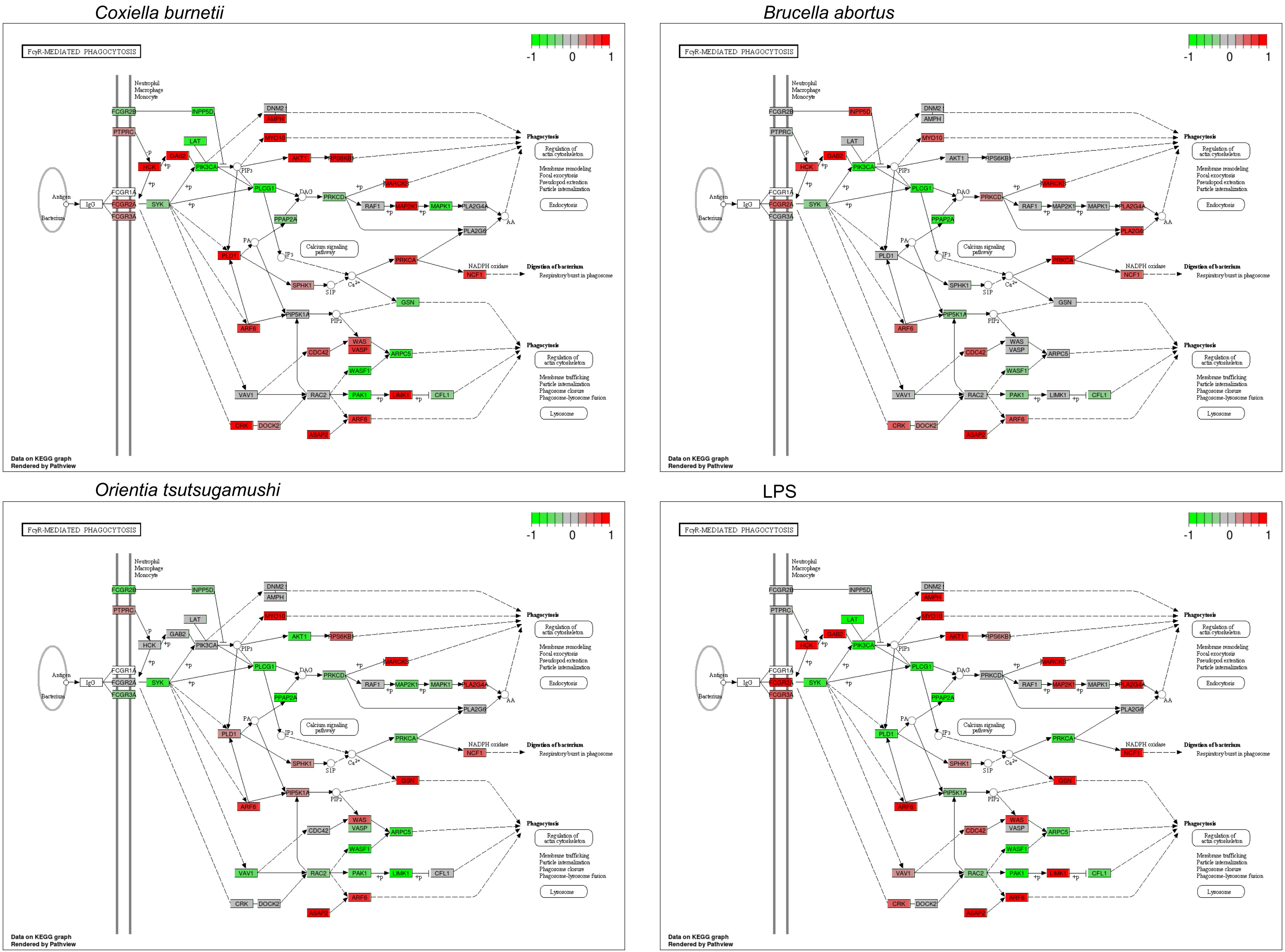
Coxiella burnetii
Brucella abortus
Orientia tsutsugamushi
LPS
FcγR-MEDIATED PHAGOCYTOSIS
-1 0 1
Neutrophil
Macrophage
Monocyte
Antigen
Bacterium
IgG
FCGR2B
PTPRC
FCGR1A
FCGR2A
FCGR3A
INPP5D
LAT
HCK
GAB2
PIK3CA
SYK
DNM2
AMPH
MYO10
AKT1
RPS6KB
PLCG1
DAG
PRKCD
MARCKS
RAF1
MAP2K1
MAPK1
PLA2G4A
AA
PPAP2A
PLA2G6
PA
PLD1
IP3
Calcium signaling pathway
PRKCA
NADPH oxidase
NCF1
SPHK1
S1P
Ca2+
GSN
ARF6
PIP5K1A
PIP2
PIP3
WAS
VASP
CDC42
ARPC5
WASF1
VAV1
RAC2
PAK1
LIMK1
CFL1
CRK
DOCK2
ASAP2
Phagocytosis
Regulation of actin cytoskeleton
Membrane remodeling
Focal exocytosis
Pseudopod extention
Particle internalization
Endocytosis
Digestion of bacterium
Respiratory burst in phagosome
Membrane trafficking
Phagosome closure
Phagosome-lysosome fusion
Lysosome
Data on KEGG graph
Rendered by Pathview
